# Anatomical and molecular characterization of parvalbumin-cholecystokinin co-expressing inhibitory interneurons: implications for neuropsychiatric conditions

**DOI:** 10.1038/s41380-023-02153-5

**Published:** 2023-07-13

**Authors:** Steven F. Grieco, Kevin G. Johnston, Pan Gao, B. Maximiliano Garduño, Bryan Tang, Elsie Yi, Yanjun Sun, Gregory D. Horwitz, Zhaoxia Yu, Todd C. Holmes, Xiangmin Xu

**Affiliations:** 1 Department of Anatomy and Neurobiology, School of Medicine, University of California, Irvine, CA 92697 USA; 2 Center for Neural Circuit Mapping, University of California, Irvine, CA 92697 USA; 3 Department of Mathematics, School of Physical Sciences, University of California, Irvine, CA 92697 USA; 4 Department of Physiology and Biophysics, University of Washington, Seattle, WA 98195 USA; 5 Department of Statistics, Donald Bren School of Information and Computer Sciences, University of California, Irvine, CA 92697 USA; 6 Department of Physiology and Biophysics, School of Medicine, University of California, Irvine, CA 92697 USA; 7 Department of Microbiology and Molecular Genetics, University of California, Irvine, CA 92697 USA; 8 Department of Biomedical Engineering, University of California, Irvine, CA 92697 USA; 9 Department of Computer Science, University of California, Irvine, CA 92697 USA

**Keywords:** Neuroscience, Psychiatric disorders

## Abstract

Inhibitory interneurons are crucial to brain function and their dysfunction is implicated in neuropsychiatric conditions. Emerging evidence indicates that cholecystokinin (CCK)-expressing interneurons (CCK+) are highly heterogenous. We find that a large subset of parvalbumin-expressing (PV+) interneurons express CCK strongly; between 40 and 56% of PV+ interneurons in mouse hippocampal CA1 express CCK. Primate interneurons also exhibit substantial PV/CCK co-expression. Mouse PV+/CCK+ and PV+/CCK- cells show distinguishable electrophysiological and molecular characteristics. Analysis of single nuclei RNA-seq and ATAC-seq data shows that PV+/CCK+ cells are a subset of PV+ cells, not of synuclein gamma positive (SNCG+) cells, and that they strongly express oxidative phosphorylation (OXPHOS) genes. We find that mitochondrial complex I and IV-associated OXPHOS gene expression is strongly correlated with CCK expression in PV+ interneurons at both the transcriptomic and protein levels. Both PV+ interneurons and dysregulation of OXPHOS processes are implicated in neuropsychiatric conditions, including autism spectrum (ASD) disorder and schizophrenia (SCZ). Analysis of human brain samples from patients with these conditions shows alterations in OXPHOS gene expression. Together these data reveal important molecular characteristics of PV-CCK co-expressing interneurons and support their implication in neuropsychiatric conditions.

## Introduction

GABAergic inhibitory interneurons comprise ~10–20% of all neurons in the brain, and contribute to the regulation of synaptic transmission, network oscillations and neural plasticity [1–3]. This diversity of interneurons is crucial to brain function, and their dysfunction may result in neuropsychiatric conditions such as schizophrenia (SCZ), autism spectrum disorder (ASD), or bipolar disorder (BD) [4]. For example, fast-spiking (FS) parvalbumin expressing (PV+) interneurons have been implicated in neuropsychiatric conditions due to their involvement in cortical oscillations and due to their high metabolic needs [5, 6]. Thus, it is imperative to study interneuron subclasses and types which are distinguished by their molecular characteristics. Previously, parsing these molecular characteristics has been difficult due to a lack of molecular tools [7, 8]. While different interneuron subclasses were first identified morphologically by Cajal over 100 years ago, genetic labeling and manipulation using transgenic mice now allows for further identification of subclasses and types and further characterization [9–13]. Neurochemical markers including parvalbumin (PV), calretinin (CR), somatostatin (SST), vasoactive intestinal peptide (VIP), neuropeptide Y (NPY), cholecystokinin (CCK), and others, are typically used to label cortical interneurons [9, 10, 14, 15]. Co-expression of different peptidergic neurochemical markers appears to extend the functional repertoire of interneurons. For example, some interneurons co-express SST and CR, and this dual neurochemical expression gives rise to unique properties [14].

Cholecystokinin (CCK) was initially characterized as a gastrointestinal peptide and was subsequently determined to be one of the most abundant neuropeptides in the central nervous system (CNS) [16]. It is synthesized as a preprohormone of 115 amino acids that can be enzymatically cleaved into multiple isoforms. The most abundant isoform in the CNS is the sulfated octapeptide CCK-8S which is expressed at high levels in the hippocampus, amygdala, septum and hypothalamus [17]. CCK is present at high levels in interneurons but is also expressed in excitatory pyramidal cells [18, 19]. Further evidence suggests that CCK-expressing interneurons (CCK+) are heterogenous, as expression of CCK is relatively promiscuous [20]. The consequence of this in each interneuron subclass is not yet understood.

To determine how CCK expression extends the repertoire of interneurons, we began by studying CCK co-expression in calcium binding protein parvalbumin (PV)-expressing interneurons, as they are one of the most abundant interneuron subclasses in the brain. PV+ fast-spiking basket cells (FSBCs) and CCK+ regular-spiking basket cells (RSBCs) are sources of perisomatic inhibition [20, 21]. PV+ interneurons are distinguished from most interneurons by their fast-spiking rates, intrinsic electrical and synaptic properties, and their role in controlling the precise timing of network oscillations. They are also very strongly implicated in a host of neuropsychiatric conditions [22]. Recent studies and databases show that PV+ interneurons express CCK at the mRNA level in both mouse and human brain [23–27].

In the present study we characterize PV+ interneurons that express CCK using an array of molecular approaches. Our transgenic approach uses PV and CCK-driver lines along with the Dlx5/6-driver line that restricts labeling to GABAergic cells and excludes labeling of excitatory neurons. We find that CCK&Dlx5/6 driven expression overlaps heavily with PV expression in GABAergic interneurons in mouse hippocampus and neocortex. We extend this finding to monkey hippocampus and find PV/CCK co-expression. For most of our subsequent work, we focus on the mouse hippocampus because of our extensive prior work there and because of its regular anatomical and neuronal structure. We find that ~40–56% of PV+ interneurons co-express CCK in mouse hippocampal region CA1. We then find distinct features in the comparative electrophysiology, transcriptomes, and chromatin characteristics of PV+/CCK+ and PV+/CCK- interneurons, which is corroborated by data mining from other groups. PV+/CCK+ interneurons strongly express oxidative phosphorylation (OXPHOS) genes, whereas PV+/CCK- interneurons do not, this relationship between CCK and OXPHOS gene expression is strongest in PV+ interneurons, as compared to other interneuron subclasses. We find that cytochrome c oxidase subunit 6A2 (COX6A2, a mitochondrial complex IV gene known to be expressed in PV+ interneurons), is strongly expressed by PV+/CCK+ interneurons. As PV+ interneuron dysfunction and dysregulation of OXPHOS processes are implicated in neuropsychiatric conditions, we analyze RNA expression in brain samples from human patients with these conditions. This analysis shows that alterations in OXPHOS gene expression are present in patients with autism spectrum disorder (ASD) or schizophrenia (SCZ). Together, our data reveal important molecular characteristics of PV-CCK co-expressing interneurons that implicate them in neuropsychiatric conditions.

## Results

### Genetic targeting of CCK+ interneurons restricted by the Dlx5/6 driver line in mouse hippocampus and neocortex

**Fig. 1:**
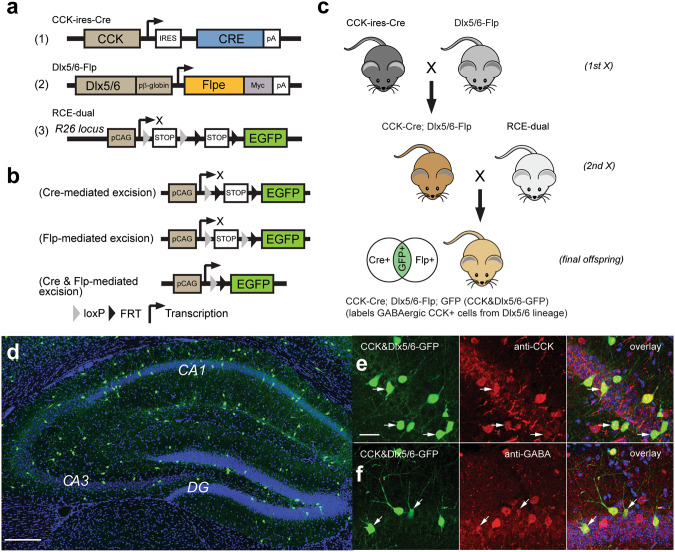
Intersectional strategy for genetic targeting of CCK+ GABAergic inhibitory interneurons in mouse hippocampus. **a** Schematic maps of the transgene structures in CCK-ires-Cre [29], Dlx5/6-Flp [30], and RCE-dual [31] (Cre and Flp dual reporter; R26R-CAG-LSL-FSF-EGFP) mice. **b** An illustration of recombinase-mediated genomic excisions. Cre recombinase is expressed in CCK+ cells by the CCK-ires-Cre driver line, while Flp recombinase is expressed in GABAergic inhibitory interneurons in the Dlx5/6-Flp driver line. In the presence of Cre and Flp recombinases, either simultaneously or successively, the STOP insert is excised via dual Cre/loxP and Flp/FRT recombinations, allowing for GFP expression in (CCK&Dlx5/6)+ GABAergic interneurons. **c** Crossbreeding strategy of transgenic mice in which their final offspring (labeled as CCK&Dlx5/6-GFP) express GFP only in (CCK&Dlx5/6)+ GABAergic inhibitory interneurons. CCK-ires-Cre mice are crossed with Dlx5/6-Flp mice. The progeny of this first cross are then crossed with the RCE-dual reporter mice. The progeny of this second cross constitute the final offspring, the CCK&Dlx5/6-GFP mice, which contain the CCK-ires-Cre, Dlx5/6-Flp, and RCE-dual transgenes. **d** A representative confocal image showing the distribution of GFP-expressing neurons (green) throughout the hippocampus in a coronal section from a CCK&Dlx5/6-GFP mouse. DAPI staining shown in blue. Scale bar is 200 µm. **e** Anti-CCK immunostaining (red) shows that a majority of the GFP-expressing cells (green) in the CCK&Dlx5/6-GFP mouse line are immunopositive for CCK (yellow), as shown in the overlay panel. Scale bar is 20 µm. **f** Anti-GABA immunostaining (red) shows that nearly all the GFP-expressing cells (green) in the CCK&Dlx5/6-GFP mouse line are immunopositive for GABA (yellow), as shown in the overlay panel. Arrows in (**e**) and (**f**) point to representative cells in each set. The scale bar in (**e**) applies to both (**e**) and (**f**). Please see Supplementary Table 1 for the percentages of CCK and GABA immunopositive CCK&Dlx5/6-GFP-expressing neurons relative to total CCK&Dlx5/6-GFP-expressing neurons in all lamina of the major structures (CA1, CA3, DG) of the hippocampus.

CCK isoforms and their preprohormone can be expressed in excitatory neurons in addition to GABAergic interneurons [28]. In order to target CCK+ inhibitory interneurons only, we employed an intersectional genetic strategy by simultaneous co-expression of two recombinases in crosses of the CCK-ires-Cre and Dlx5/6-Flp mouse driver lines [29, 30] (Fig. 1a, c). Cre recombinase is expressed in CCK+ cells by the CCK-ires-Cre driver line, while Flp recombinase is expressed in GABAergic interneurons by the Dlx5/6-Flp driver line. Thus, the Dlx5/6 driver line restricts expression to Dlx5/6+ GABAergic inhibitory neurons and excludes expression in excitatory neurons. This intersectional cross results in progeny that carry both Cre and Flp (CCK-Cre: Dlx5/6-Flp), and thus isolates Cre and Flp expression to CCK+ GABAergic interneurons restricted by the Dlx5/6 driver line (CCK&Dlx5/6). To visualize CCK+ GABAergic interneurons, CCK-Cre: Dlx5/6-Flp animals were crossed with the RCE-dual reporter line (R26R-CAG-LSL-FSF-EGFP) [31]. EGFP is expressed only in cells where both Cre and Flp are expressed, thus mediating dual Cre/loxP and Flp/FRT recombinations (Fig. 1b, c). Mouse hippocampal CCK+ GABAergic interneurons labeled by this genetic scheme (Fig. 1c) are marked by GFP expression (Fig. 1d) and exclude CCK+ excitatory neurons.

We validated the specificity of GFP labeling in the CCK+ cells restricted by the Dlx5/6 driver line using CCK and GABA immunocytochemical staining in hippocampus (Fig. 1e, f). Across hippocampal regions CA1, CA3, and dentate gyrus (DG), the co-identity ratio of CCK immunopositive CCK&Dlx5/6-GFP-labeled cells to all CCK&Dlx5/6-GFP-labeled cells is 96.7% (total 2489 cells counted, *n* = 3; Supplementary Table 1). Similarly, the overall ratio of GABA immunopositive CCK&Dlx5/6-GFP-labeled cells to all CCK&Dlx5/6-GFP-labeled cells is 95.8% (total 2723 cells counted, *n* = 3; Supplementary Table 1). Thus, we confirm the specificity of GFP labeling in the CCK+ cells in mouse hippocampus restricted by the Dlx5/6 driver line for CCK+ GABAergic interneurons, corroborating results from two previous studies that used a similar Dlx5/6 driver line approach [23, 32]. We find that CCK+ cells restricted by the Dlx5/6 driver line (labeled by GFP) make up ~30% of all GABA immunopositive cells, which is supported by a previous study that used a similar intersectional transgenic approach and showed that CCK+ interneurons make up ~20–30% of all GABAergic cells [32]. Another recent study used the intersectional transgenic approach and reported that ~78% of all GABAergic CCK immunopositive cells are labeled in the CCK&Dlx5/6-GFP mouse cortex [23].

### A significant subset of mouse hippocampal interneurons are CCK+ and PV+

**Fig. 2:**
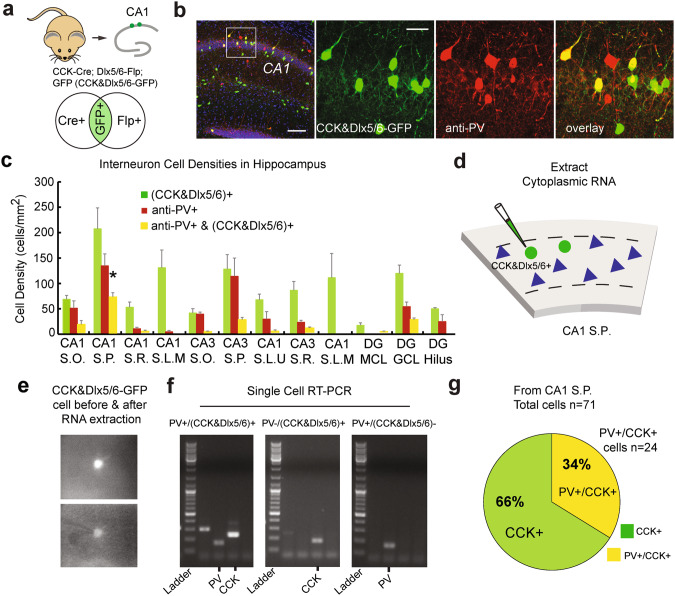
Co-localization of CCK+ and PV+ inhibitory interneurons in mouse hippocampus. **a**–**c** Co-localization of (CCK&Dlx5/6)+ and PV+ inhibitory interneurons in mouse hippocampus using immunostaining. **a** A cartoon schematic depicting the CCK&Dlx5/6-GFP mouse line containing the CCK-ires-Cre, Dlx5/6-Flp, and RCE-dual transgenes. The Venn diagram shows that GFP-expressing cells are (CCK&Dlx5/6)+ GABAergic interneurons that are Cre+ and Flp+. **b** (left panel) A low magnification (4x) confocal image showing the distribution of GFP-expressing neurons (green) throughout the CA1 region of hippocampus in a coronal section from the CCK&Dlx5/6-GFP mouse. Anti-PV immunostaining signal is shown as well (red). DAPI staining shown in blue. Scale bar is 100 µm. The other panels show higher magnification images (10x) of the white box region in the first panel, that are also digitally enlarged. Anti-PV immunostaining (red) shows that many of the GFP-expressing cells (green) in the CCK&Dlx5/6-GFP mouse line are immunopositive for PV (yellow), as shown in the overlay panel in the fourth column panel. DAPI staining is shown in blue. The scale bar in the second panel applies to the second, third, and the fourth columns. The scale bar is 20 µm. **c** A bar plot of the average densities (cells/mm^2^) of (CCK&Dlx5/6)+ (green), PV+ (red), and PV+/ (CCK&Dlx5/6)+ (yellow) interneurons in all major regions (CA1, CA3, DG) and sublamina of the CCK&Dlx5/6-GFP mouse hippocampus. The CA1 S.P. region had the highest cell density (cells/mm^2^) for (CCK&Dlx5/6)+, PV+ and PV+/(CCK&Dlx5/6)+ interneurons as compared to the rest of the hippocampus. Please see Supplementary Tables 2–4 for numerical data corresponding to this histogram. Co-localization of (CCK&Dlx5/6)+ and PV+ inhibitory interneurons in mouse hippocampus using RT-PCR. **d** A cartoon schematic depicting the extraction of the cytoplasmic contents of individual CA1 hippocampal interneurons from CCK&Dlx5/6-GFP mice. **e** The cytoplasmic contents of individual GFP+ cells from live ex vivo hippocampal sections were aspirated using a glass micropipette enabling visualization, using a high-magnification (63×) fluorescent microscope, before (top panel) and after (bottom panel) extraction of cytoplasmic contents. The contents of each individual cell were then transferred to a single tube containing buffers for downstream applications. **f** Using custom primers (see Methods) specific for mouse *Cck* and *Pvalb* mRNA, RT cDNA was then amplified by PCR using a thermocycler. PCR products were then segregated on an agarose gel, and stained with ethidium bromide allowing the visualization and determination of *Cck* and/or *Pvalb* mRNA expression from each cell. Representative cells PV+/(CCK&Dlx5/6)+, PV-/(CCK&Dlx5/6)+, and PV+/(CCK&Dlx5/6)- are shown with a DNA low molecular weight ladder on the left of each gel. A *Pvalb* mRNA-expressing cell is shown as a positive control. **g** Of all individual cells analyzed (*n* = 71), only 66% expressed *Cck* mRNA (PV-/CCK+)(green) and not *Pvalb* mRNA, and 34% expressed *Cck* and *Pvalb* mRNA (PV+/CCK+)(yellow).

To measure co-localization of CCK+ cells restricted by the Dlx5/6 driver line with PV+ interneurons in mouse hippocampus, we performed anti-PV immunolabelling of CCK+ cells restricted by the Dlx5/6 driver line (Fig. 2a, b). We also measured co-localization of CCK+ cells restricted by the Dlx5/6 driver line with other major neurochemical interneuron marks, revealing the relative frequency of the co-localization of CCK+ cells restricted by the Dlx5/6 driver line with PV+ interneurons compared to other interneuron markers (Supplementary Figs. 1, 2; Supplementary Tables 2–4). PV immunostaining of CCK+ cells restricted by the Dlx5/6 driver line in the somatosensory (S1) barrel cortex region also reveals the co-localization of CCK+ cells restricted by the Dlx5/6 driver line with PV+ interneurons (Supplementary Fig. 3). In the S1 cortical layers L1–6, ~48% of PV+ interneurons and ~37% of CCK+ cells restricted by the Dlx5/6 driver line are both PV+ and CCK+.

We quantified the prevalence of PV+ interneurons, and the degree of their co-localization with CCK&Dlx5/6-GFP signal in the sub-regions and different lamina of the mouse hippocampus. Both CCK+ cells restricted by the Dlx5/6 driver line and PV+ interneurons are concentrated among the hippocampal principal cell layers (Fig. 2c; Supplementary Tables 3, 4). Fewer PV+ interneurons are located in the CA1 and CA3 stratum lacunosum moleculare (SLM) layers and the DG molecular cell layer (ML). Surprisingly, many interneurons are (CCK&Dlx5/6)+ and PV+, and are concentrated in the CA1 and CA3 pyramidal cell layers and the DG granule layer with cell densities of 63.3 ± 7.2/mm^2^, 22.9 ± 4.2/mm^2^, and 22.7 ± 2.8/mm^2^ (mean ± SD), respectively. In the CA1 stratum pyramidale (SP) layer, nearly half (48.1 ± 7.8%) of PV+ interneurons are (CCK&Dlx5/6)+. PV+ interneuron density in CA1 SP is 134.6 ± 23.5/mm^2^, CCK+ interneuron density in CA1 SP is 207.5 ± 41.2/mm^2^ (Fig. 2c; Supplementary Tables 3, 4). Thus, there are many PV+ and (CCK&Dlx5/6)+ co-expressing interneurons in mouse hippocampus, primarily located in the principal cell layer.

To confirm these findings, we use single-cell RT-PCR measuring *Pvalb* and *Cck* mRNA expression levels in the cytoplasmic contents of GFP+ cells of the CA1 SP layer from CCK&Dlx5/6-GFP mice (Fig. 2d, e). The RT-PCR results are in close agreement with our immunocytochemical staining results: ~34% of all (CCK&Dlx5/6)+ interneurons in CA1 SP also express *Pvalb* mRNA (Fig. 2f, g). These findings show that a substantial subset of interneurons co-express *Cck* and *Pvalb* mRNA in mouse hippocampus. To further confirm these findings in PV-Cre derived mice, we use single nuclei RNA sequencing (snRNA-seq) of PV+ interneurons to determine *Cck* mRNA expression levels in this population from the entire hippocampus, not just CA1. We use FANS (fluorescence-activated nuclei sorting) sorted GFP+ nuclei from PV-Cre; cSUN1 mice which express a GFP-tagged nuclear membrane in a PV-cell-specific manner [33]. Analysis of this data set reveals that a significant percentage of PV-expressing interneurons in total mouse hippocampus also express *Cck* mRNA, which agrees with our immunostaining results of the entire hippocampus (Supplementary Fig. 4). Thus, we show the prevalence of PV+ and CCK+ co-expressing interneurons in mouse hippocampus using multiple complementary methodologies.

### In contrast to rat, CCK and PV co-express in mouse and macaque hippocampal inhibitory interneurons

The literature consensus from rat data is that CCK+ and PV+ interneurons are two distinct non-overlapping interneuron subclasses [16–19, 34]. Based on our findings and the recent literature that supports the idea that a subset of PV+ interneurons co-express CCK in mouse hippocampus, we decided to revisit this issue in rat. Using validated antibodies (see Methods and Supplementary Table 6 for details) for the CCK and PV proteins, we confirm earlier findings that CCK+ and PV+ interneurons do not show overlapping expression in rat hippocampus (Supplementary Fig. 5a–c). In contrast, using the same antibodies, we find that PV and CCK expression overlaps in a subset of interneurons in the mouse hippocampus (Supplementary Fig. 5d–f). For further cross-species comparison, we perform the same immunostaining procedure using the same antibodies on pig-tailed macaque monkey hippocampal sections. Like mouse hippocampus, pig-tailed macaque monkey hippocampus has a population of interneurons that co-express CCK and PV (Supplementary Fig. 5g–i); 34% of PV+ interneurons are CCK+ (*n* = 3 macaque hippocampi, 138 PV+ and CCK+ co-expressing cells out of 403 PV+ interneurons).

### Genetic targeting and characterization of PV+/CCK+ inhibitory interneurons in mouse hippocampus

**Fig. 3:**
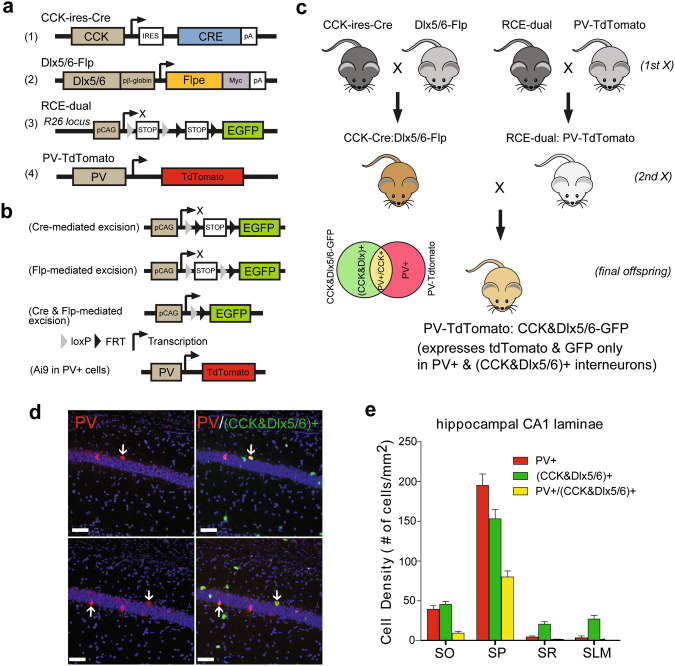
Intersectional strategy for genetic targeting of PV+/CCK+GABA-ergic inhibitory interneurons in mouse hippocampus. **a** Schematic maps of the transgene structures in CCK-ires-Cre, Dlx5/6-Flp, RCE-dual (Cre and Flp dual reporter; R26R-CAG-LSL-FSF-EGFP), and PV-tdTomato mice. **b** An illustration of recombinase-mediated genomic excisions and PV-mediated expression of tdTomato. Cre recombinase is expressed in CCK+ cells by the CCK-ires-Cre driver line, while Flp recombinase is expressed in GABAergic inhibitory interneurons in the Dlx5/6-Flp driver line. In the presence of Cre and Flp recombinases, the STOP insert is excised via dual Cre/loxP and Flp/FRT recombinations, allowing for EGFP expression in CCK+ GABAergic interneurons. In PV-tdTomato mice fluorescent reporter is expressed in PV+ cells. **c** Crossbreeding strategy of transgenic mice in which their final offspring (labeled as PV-tdTomato; CCK&Dlx5/6-GFP) express tdTomato and GFP only in PV+/(CCK&Dlx5/6)+ GABAergic inhibitory interneurons. CCK-ires-Cre mice are crossed with Dlx5/6-Flp mice. The progeny of this first cross are then crossed with the progeny of cross between RCE-dual reporter mice and PV-tdTomato mice. The progeny of this second cross constitutes the final offspring, the PV-tdTomato; CCK&Dlx5/6-GFP mice, which contain the CCK-ires-Cre, Dlx5/6-Flp, RCE-dual, and PV-tdTomato transgenes. (**d**, left panels) Confocal images showing the distribution of tdTomato-expressing PV+ cells in the CA1 region of hippocampus in a coronal section from a PV-tdTomato; CCK&Dlx5/6-GFP mouse. Only the red channel is shown. (**d**, right panels) The same sections showing the distribution of tdTomato-expressing PV+ cells (red) and (CCK&Dlx5/6)+ GABAergic interneurons (green). In this PV-tdTomato; CCK&Dlx5/6-GFP mouse, interneurons that are PV+/(CCK&Dlx5/6)+ (yellow) are indicated with arrowheads. DAPI staining is shown in blue. The scale bars are 50 µm. **e** A bar plot of the average densities (cells/mm^2^) of (CCK&Dlx5/6)+ (green), PV+ (red), and PV+/(CCK&Dlx5/6)+ (yellow) cells in all sublamina (SO, SP, SR, SLM) of CA1 in the PV-tdTomato; CCK&Dlx5/6-GFP mouse hippocampus. The CA1 S.P. region has the highest cell density (cells/mm^2^) for (CCK&Dlx5/6)+, PV+ and PV+/(CCK&Dlx5/6)+ interneurons as compared to the rest of the hippocampus. Please see Supplementary Table 5 for numerical data corresponding to this bar plot.

To study PV+ co-expression with CCK+ cells restricted by the Dlx5/6 driver line in greater detail, we build on the approach described in Fig. 1 to specifically label PV+ and (CCK&Dlx5/6)+ neurons that express different combinations of fluorescent proteins. We achieve this by adding an additional mouse line cross (PV-tdTomato) to generate animals carrying four transgenes: PV-tdTomato, CCK-Cre, Dlx5/6-Flp, and the RCE dual reporter (Fig. 3a–c). The resultant cross, which we term “PV-tdTomato; CCK&Dlx5/6-GFP”, enables intersectional dual-labeling of GABAergic PV+/(CCK&Dlx5/6)+ interneurons in the hippocampus and neocortex using two distinct genetically encoded fluorescent markers.

We characterized the multi-labeled mouse model by examining the relative co-localization of PV+ and (CCK&Dlx5/6)+ interneuron populations in hippocampus. We find that both PV+ (tdTomato) and (CCK&Dlx5/6)+ (GFP) interneuron populations are distributed throughout CA1, CA3, and the DG regions in the PV-tdTomato; CCK&Dlx5/6-GFP mouse. High-power confocal images of the CA1 region again show the existence of PV+/(CCK&Dlx5/6)+ cell populations (Fig. 3d). We then quantify the CA1 laminar distributions of PV+ cells that co-express CCK as restricted by the Dlx5/6 driver line. Similar to our results reported in Fig. 2, we verify that mouse CA1 layer SP contains the highest density of PV+ cells that co-express CCK+ restricted by the Dlx5/6 driver line, as compared to the other CA1 layers (195.5 ± 47.0/mm^2^, mean ± SD, 1278 cells sampled from at least 3 mice) (Fig. 3e; Supplementary Table 5). We also find a significant proportion of PV+/CCK+ interneurons present in the somatosensory (S1) barrel cortex of the PV-tdTomato; CCK&Dlx5/6-GFP mouse (Supplementary Table 5). This result using the PV-tdTomato; CCK&Dlx5/6-GFP mouse corresponds well with our findings in Supplementary Fig. 3 where the anti-PV immunostaining of the CCK&Dlx5/6-GFP mouse S1 region reveals a population of PV+ and CCK+ co-expressing interneurons.

We then examined whether there are differences in the morphological characteristics of PV+/(CCK&Dlx5/6)+ and PV+/(CCK&Dlx5/6)- interneurons using the dual-labeled PV-tdTomato; CCK&Dlx5/6-GFP mouse. In acutely prepared cortical slices, PV+/(CCK&Dlx5/6)+ (*n* = 22) and PV+/(CCK&Dlx5/6)- (*n* = 13) interneurons located in the hippocampal CA1 pyramidal cell layer were recorded and filled with biocytin for *post-hoc* morphological analysis (Supplementary Fig. 6a–i). We quantify the dendritic length, area and the somatic volume of PV+/(CCK&Dlx5/6)+ and PV+/(CCK&Dlx5/6)- interneurons, along with Sholl analysis of dendritic arbor complexity. For all somatodendritic variables assessed, PV+/(CCK&Dlx5/6)+ and PV+/(CCK&Dlx5/6)- interneurons were similar (Supplementary Fig. 6m–p). We also provide volumetric confocal (Supplementary Videos 1–3) and light-sheet (Supplementary Video 4) imaging scans highlighting the characteristics of CA1 PV+/(CCK&Dlx5/6)+ and PV+/(CCK&Dlx5/6)- interneurons using the dual-labeled PV-tdTomato; CCK&Dlx5/6-GFP mouse (Supplementary Videos 1–4).

**Fig. 4:**
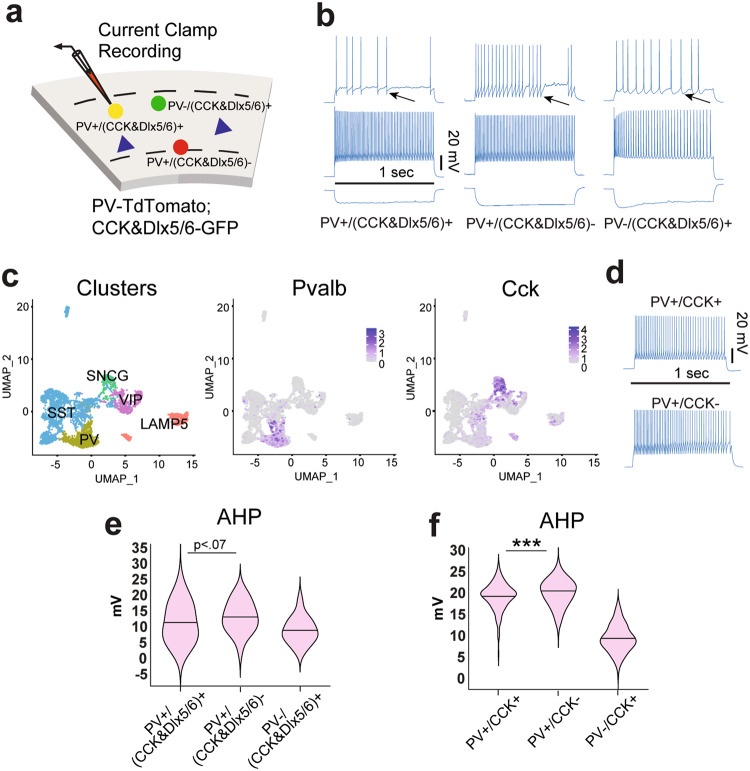
Comparison of electrophysiology for PV+/CCK+ and PV+/CCK- GABAergic inhibitory interneurons in mouse. **a**, **b**, **e** Electrophysiological characterization of the PV-tdTomato; CCK&Dlx5/6-GFP mouse harboring the CCK-ires-Cre, Dlx5/6-Flp, RCE-dual, and PV-tdTomato transgenes. **a** An illustration of current clamp recording from CA1 hippocampal neurons that are either PV+/(CCK&Dlx5/6)+ (yellow), PV-/(CCK&Dlx5/6)+ (green) or PV+/(CCK&Dlx5/6)- (red). Pyramidal excitatory cells are depicted as blue triangles. **b** Current clamp electrophysiological recording from acute brain slices containing region CA1. Representative voltage traces for PV+/(CCK&Dlx5/6)+, PV+/(CCK&Dlx5/6)- or PV-/(CCK&Dlx5/6)+ neurons in response to depolarizing current steps. The vertical scale bar is 20 mV. The horizontal scale bar is 1 s. **c**, **d**, **f** Patch-seq data from adult mouse cortical interneurons made publically available from the Allen Institute. **c** SnRNA-seq data were processed as follows. Cells exhibiting an extremely high or low number of features were eliminated. Count matrices were log-normalized and scaled. Highly variable genes were identified and used as features for PCA dimensionality reduction. Next, anchors were identified and the data were integrated via the Seurat framework. The data were clustered and projected to two dimensions via UMAP for visualization. The cell subclasses were identified via expression of interneuron gene markers (*Sst, Pvalb, Vip, Lamp5, and Sncg*) (**c**, Left). (Pvalb)* Pvalb* gene expression shows strong localization to the PV subclass (**c**, middle). (Cck) *Cck* gene expression is exhibited in both SNCG+ and PV+ interneurons (**c**, right). For electrophysiology data are further analyzed through the following pipeline. PV+ interneurons were identified as PV+/CCK+ if *Cck* expression is above the 75th percentile, and PV+/CCK- if *Cck* expression is below the 25th percentile. **d** Current clamp electrophysiological recording from acute brain slices containing visual cortex. Representative voltage traces for PV+/CCK+, or PV+/CCK- interneurons in response to depolarizing current steps. The vertical scale bar is 20 mV. The horizontal scale bar is 1 s. **e** Summary violin plot of the electrophysiological property “afterhyperpolarization” (AHP) (value of negative peak reached from threshold) from PV+/(CCK&Dlx5/6)+ (*n* = 24), PV+/(CCK&Dlx5/6)- (*n* = 19), and PV-/(CCK&Dlx5/6)+ (*n* = 15) interneurons. The violins show data with the mean line in the middle. The top and bottom of the plots are the minima and maxima (Kruskal-wallis test: overall *p* = 0.0007. Mann Whitney test: PV+/(CCK&Dlx5/6)+ versus PV+/(CCK&Dlx5/6)-, *p* = 0.0637). **f** Summary violin plot of afterhyperpolarization (AHP) peaks from PV+/CCK+ (*n* = 178), PV+/CCK- (*n* = 169) and PV-/CCK+ (*n* = 198) interneurons from the Allen Institute data set. The violins show data with the mean line in the middle. The top and bottom of the plots are the minima and maxima. PV+/CCK- interneurons have significantly larger afterhyperpolarization (AHP) amplitudes compared to PV+/CCK+ interneurons (One-way ANOVA: overall *p* < 6.72 e-96. Bonferroni multiple comparisons: PV+/CCK+ versus PV+/CCK-, *p* = 1.19 e-6).

We then characterized the electrophysiological properties of PV+/(CCK&Dlx5/6)+, and PV+/(CCK&Dlx5/6)- interneurons by recording from them in the CA1 SP layer with a whole cell current clamp configuration using the multi-labeled PV-tdTomato; CCK&Dlx5/6-GFP mouse (Fig. 4; Supplementary Fig. 7). Step current injection-induced action potentials were analyzed from 24 PV+/(CCK&Dlx5/6)+ and 19 PV+/(CCK&Dlx5/6)- cells (Supplementary Fig. 7). In CA1, the mean afterhyperpolarization (AHP) values (the value of the negative peak reached from threshold) of PV+/(CCK&Dlx5/6)- cells (13.64mV) and PV+/(CCK&Dlx5/6)+ cells (10.20 mV) show a trend of difference (Fig. 4e). To support our finding, we data-mined published Patch-seq data publicly available from the Allen Institute [26]. This data set consists of 4,270 cortical GABAergic interneurons from adult mouse visual cortex (V1). We identify 724 PV+ interneurons and based on dimensionality reduction CCK+ cells show clear overlap (Fig. 4c). We characterize PV+/CCK+ and PV+/CCK- interneurons and similar to trends from our recordings, we find that the afterhyperpolarization (AHP) values significantly differ between PV+/CCK+ (*n* = 178) and PV+/CCK- (n = 169) interneurons (*p* = 1.19 e-6) (Fig. 4f). These results show that there are electrophysiological features that may distinguish PV+ interneurons that strongly express CCK versus those that do not.

We then characterized behavioral aspects of PV+/CCK+ interneurons. Previous work shows that hippocampal PV+ interneurons are important for hippocampus-dependent memory formation and are impaired in psychiatric conditions [35–38]. We tested if CA1 PV+/CCK+ cells contribute functionally to these processes using DREADDs (designer receptors exclusively activated by designer drugs)-mediated inactivation [39]. To express hM4Di in CA1 PV+/CCK+ cells, we crossed CCK-ires-Cre mice with Pvalb-2A-Flp mice, then Cre and Flp dual controlled AAV (AAV-Syn-Con/Fon-hM4Di-mCherry-WPRE) was injected into CA1 of the progeny. The hM4Di-expressing PV+/CCK+ cells are then acutely inactivated by systemic injection of hM4Di ligand clozapine-N-oxide (CNO) (Supplementary Fig. 8a, b).

To determine if PV+/CCK+ interneuron inactivation affects object-location memory (OLM), mice were first allowed to explore two identical objects within a behavioral arena during a “training session”. Then, during the “test session”, the location of one of the two objects was moved. CNO was injected prior to the training session (Supplementary Fig. 8c). Mice that receive CNO display much lower object-location discrimination as shown by their reduced time spent with the moved object (Supplementary Fig. 8d, e).

CCK+ interneurons are thought to receive inputs concerning the emotional state of the animal [40–43]. We tested if CA1 PV+/CCK+ cells functionally contribute to emotion/affective behavior with a hippocampus-dependent memory component [44–46]. To determine if PV+/CCK+ interneuron inactivation affects fear renewal, mice were initially exposed to foot shocks paired with sound (fear conditioning). For two consecutive days mice were then moved to a similar context and exposed to the sound only (fear extinction). One week later normal adult mice exhibit renewed fear if they are exposed to the sound (fear renewal). CNO was injected before the fear renewal session (Supplementary Fig. 8b, f). Acute inactivation of PV+/CCK+  inhibitory interneurons in CA1 results in significantly reduced freezing behavior as compared to controls during the fear renewal session (Supplementary Fig. 8g).

### PV+/CCK+ and PV+/CCK- inhibitory interneurons are distinguished by their transcriptomic profiles

**Fig. 5:**
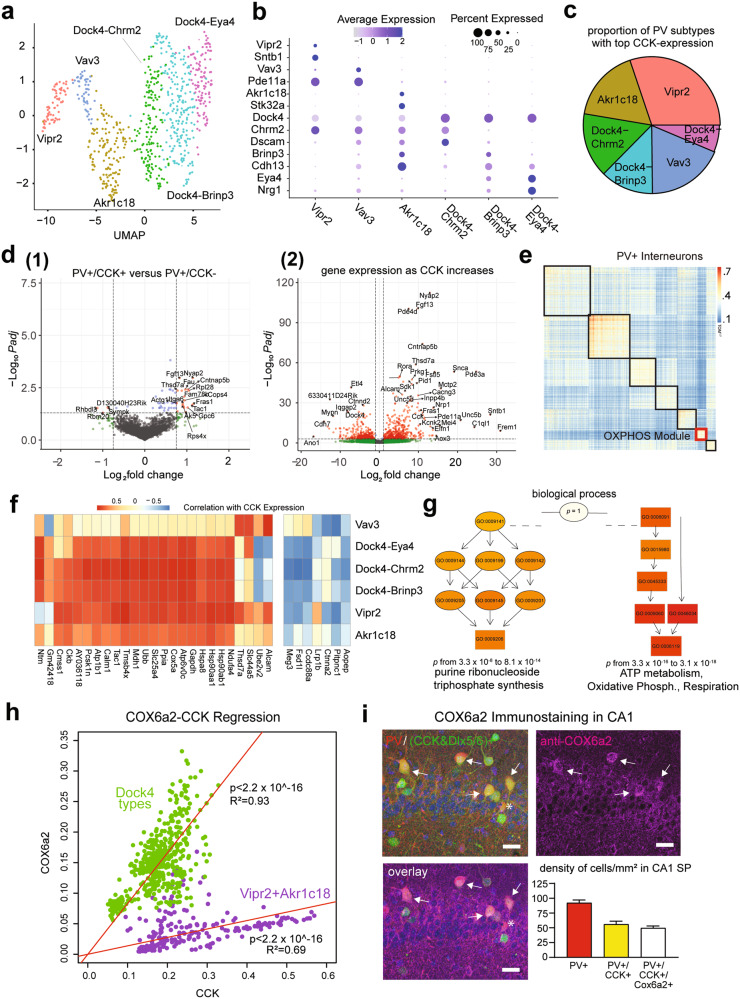
Hippocampal CCK-expressing PV+ interneurons are enriched with oxidative phosphorylation (OXPHOS) gene expression. Single-nucleus RNA-seq was performed on hippocampi from wild-type (WT) mice, and analysis was performed on data from PV+ interneurons. **a** UMAP clustering of snRNA-seq data identifies six PV+ interneuron cell types. These types were initially labeled using the Tasic et al., 2018 classification to find the Vipr2 and Akr1c18 types. The Dock4 type was split into three separate clusters, though the Dock4-Brinp3 subtype appears to be an intermediate state, with few unique markers even after restricting analysis to the Dock4 cells. The Vav3 cell type did not appear in the Tasic et al., 2018 classification or the Allen Institute’s 2021 10x Genomics dataset from mouse cortex and hippocampus, and may be an intermediate state PV+ cell type. Our stringent QC requirements decrease the likelihood that any PV+ cell type is composed primarily of doublets. **b** Dot Plot of normalized gene expression of marker genes (y-axis) in clusters (x-axis). **c** Percentage of top *Cck* expressing cells by cluster, analyzed using imputed data. **d** Volcano plots based on *Cck* expression: (1) gene expression differences in *Cck* expressing cells versus *Cck* non-expressing cells; (2) gene expression variations as a function of imputed *Cck* expression, treated as a continuous variable. **e** Gene co-expression analysis via WGCNA identified several highly co-expressed modules. Genes exhibiting co-expression less than 0.15 were removed from the visualization. The rest clustered into seven groupings, including a COX grouping, that was also highly co-expressed with *Cck*. **f** Heatmap of top ranked genes co-expressing with *Cck*, divided by cluster. Intensity indicates the correlation of imputed expression of each gene (x-axis) and *Cck* expression after imputation in each cluster (y-axis). **g** Gene ontology for a selection of top ranked genes aggregated from each method. Ontology terms divide into purine ribonucleotide triphosphate synthesis, and a combined ATP metabolism/oxidative phosphorylation term. **h** Regression plot comparing *Cox6a2* and *Cck* expression in either the PV+ cell Dock4 type (green) or the remaining PV+ cell types (purple). **i** Immunostaining of the COX6A2 protein (magenta) in CA1 from PV-tdTomato; CCK&Dlx5/6-GFP (yellow) mouse brain sections (top left, right). COX6A2 staining was robust in PV+/(CCK&Dlx5/6-GFP) (white) cells (bottom left) (scale bar = 20 µm). COX6A2 was co-localized with ~89% of PV + /(CCK&Dlx5/6)+ (white, arrows). Cell with an asterisk is an example of a PV+ cell that is COX6A2 negative. Quantification of cell densities in CA1 for COX6A2 immunopositive PV+/(CCK&Dlx5/6)+ cells (bottom right)(*n* = 3 mice). Data are presented as the mean ± S.E.M.

We characterized the transcriptomic properties of PV+/CCK+ and PV+/CCK- interneurons in wild-type (WT) adult mouse hippocampus by analyzing snRNA-seq data obtained in our laboratory from simultaneous snRNA/ATAC (10x Multiomics) (Fig. 5). We use WT mice as we did not want transgenes to affect our data. Initial analysis of hippocampal snRNA-seq verifies data quality and identifies PV+ interneurons in each dataset based on *Pvalb* and *Gad1/Gad2* co-expression (Supplementary Fig. 9a–d). Clustering results in the identification of six PV+ interneuron clusters (*n* = 737 total cells, labeled as Vipr2, Vav3, Akr1c18, and Dock4, which is divided into three subtypes). PV+ interneurons are known to be less numerous in the hippocampus compared with the neocortex [9, 12, 47], explaining the relatively low yield. We compare our data with the Allen Institute and BICCN data sets (Supplementary Fig. 9) for the derivation and comparison of taxonomies. The Vipr2 and Akr1c18 clusters are annotated based on known taxonomical markers [48], while *Dock4* is present in Vipr2- cells in the Allen’s dataset and is a strong differentiating factor for the remaining clusters in our data set. The Dock4 cell type is subdivided into three unique subtypes based on agnostic clustering results, and these subtypes are indicated by the top ranked differentially expressed gene in each (*Chrm2, Brinp3, and Eya4*). The Vav3 cluster is also annotated based on its top differentially expressed gene. The Vav3 and Dock4-Brinp3 clusters appear to be intermediate states based on the presence of fewer unique markers and their split disposition in the Allen dataset (Supplementary Fig. 9e–g) [48].

The majority (64.7%) of PV+ interneurons are part of the Dock4 cell type, which collectively with the Akr1c18 cell type (19.5%) comprise the previously reported Tac1+, PV+ interneurons [25–27, 49]. The Vipr2 and Vav3 clusters are taxonomically and transcriptomically distinct from Tac1+, PV+ interneurons (Supplementary Fig. 9c). Altogether, the identified PV+ interneuron types and their associated marker genes are largely recapitulated by the hippocampal snRNA-seq mouse data available from Allen, with most hippocampal cell types intersecting uniquely with 1–2 Allen annotated types (Supplementary Fig. 9g).

After establishing PV+ cell types and subtypes (Fig. 5a, b), we then analyzed raw transcripts of *Cck* expression in PV+ interneurons, and find that 23.3% of cells express at least one count of *Cck*. This expression percentage exceeds that of 93.5% of genes, and considering a probable transcript dropout rate of ~85% [50], corresponds well with the 40–56% rate seen previously in our co-labeled transgenic animals. After imputation using MAGIC, cells exceeding the median expression level of *Cck* are distributed across all PV+ interneurons (Fig. 5c).

We next computed differential gene expression between PV+/CCK+ and PV+/CCK- cells (“CCK+” defined as containing a single expressed *Cck* gene), as well as differential gene expression computed by considering imputed *Cck* expression as a continuous variable (Fig. 5d1,2). For continuous variation, log-fold change can be interpreted as the slope of a regression line obtained by plotting individual gene expression against imputed *Cck* expression as the dependent variable. The use of imputed *Cck* has an inflationary effect on log-fold changes, but more accurately depicts the continuous spectrum of *Cck* expression. These analyses show a significant subset of *Cck* co-regulated genes, indicative of significant variation along a continuous spectrum defined by *Cck* expression (Fig. 5d; Supplementary Fig. 10a, b, d).

We next considered those genes exhibiting the largest co-expression with *Cck*. For each cell type (and subtype), we identify the top 10% of genes co-upregulated with *Cck* (differential expression treating imputed *Cck* as a continuous variable). This identifies a group of 104 genes strongly correlated with *Cck* expression. We confirm this analysis by computing the correlation of imputed expression of these genes with *Cck* (correlation 0.65 ± 0.29, mean ± standard deviation across cells) (Fig. 5f; Supplementary Fig. 9h).

Gene ontology overexpression testing by topGO identifies two major functions associated with *Cck* co-upregulated genes: purine ribonucleoside triphosphate synthesis, and a combined ATP metabolism and oxidative phosphorylation (OXPHOS) module. OXPHOS, the mitochondrial electron transport chain consisting of complexes I-IV which generate ATP, was the top GO term for PV+/CCK+ cells based on gene expression (Fig. 5g). Using WGCNA to compute gene co-expression modules, we again identify an OXPHOS co-regulation module (Fig. 5e), which contains many genes highly correlated with *Cck* expression. Many of these are from the *Cox* (cytochrome c oxidase) or *Nduf* (NADH: ubiquinone oxidoreductase) families (complexes I and IV of the electron transport chain). These results were replicated by analysis of databases for cortical PV+ interneurons from the Allen Institute (Supplementary Fig. 11).

To validate these transcriptomic findings at the protein level, we select COX6A2, an ADP-sensitive isoform of COX6A [51, 52] that is consistently correlated with *Cck* expression in both hippocampal and cortical snRNA-seq (Fig. 5h), and is highly restricted to PV+ interneurons [53]. Immunostaining in the CA1 region of brain sections from our PV-tdTomato; CCK&Dlx5/6-GFP mouse shows that most PV+/CCK+ cells are COX6A2+ (*n* = 168 cells; ~89% positive), but most PV+/CCK- cells are not COX6A2+ (*n* = 109 cells; ~14% positive) (Fig. 5i). This immunostaining result agrees with our transcriptomic findings that *Cck* expression by PV+ cells correlate strongly with increased *Cox6a2* expression (Fig. 5h; Supplementary Fig. 11c). We also find that PV+ interneurons account for ~70% of all COX6A2+ cells. Together these results show that COX6A2 is predominantly expressed in PV+/CCK+ interneurons, and not in PV+/CCK- cells, and that PV+/CCK+ interneurons have unique molecular profiles that are verifiable at the protein level.

### PV+/CCK+ and PV+/CCK- inhibitory interneurons are distinguished by their chromatin accessibility profiles

**Fig. 6:**
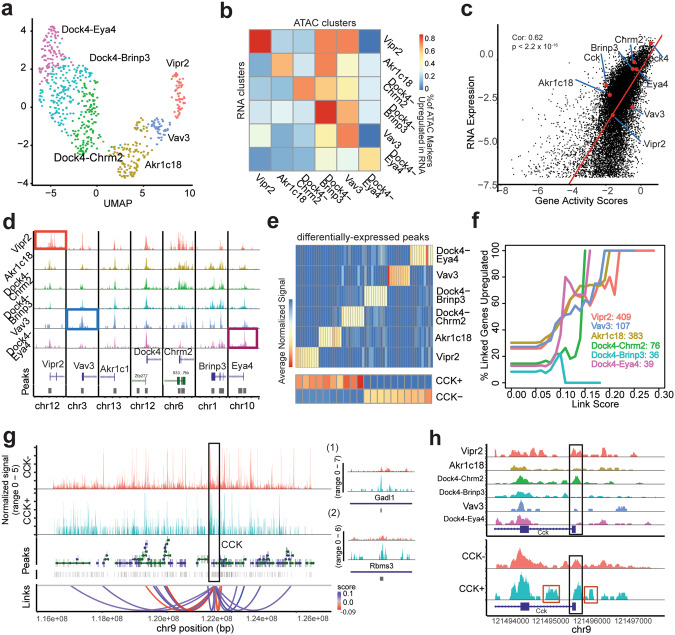
Hippocampal CCK-expressing PV+ interneurons are distinguished by their chromatin accessibility features. **a** snATAC-seq recapitulates the snRNA-seq clustering. RNA cluster labels are superimposed on the snATAC UMAP after TFIDF-SVD-UMAP dimensionality reduction. **b** Heatmap shows the percentage of differentially expressed ATAC genes (based on gene activity scores) in each cluster (x-axis) that were also differentially expressed in the RNA (y-axis). **c** Average inferred RNA expression (based on ATAC peak accessibility near TSSs) is shown for each gene, and plotted against average RNA expression for each gene. Genes of interest are shown in red. **d** Peak accessibility is shown at each marker label. Several markers (*Vipr2, Vav3, Eya4*) exhibit differential accessibility at transcription start sites (TSSs), while the remainder are consistently accessible in all cell types. **e** Identified peaks differentially accessible in the six types, as well as aggregated CCK+ vs CCK- (based on having at least one expressed *Cck* count) cells. **f** Upon performing peak to gene linkage analysis, we computed the intersection between upregulated peaks in each cell type and distal peaks determined as cis-regulatory elements. We then considered the percentage of linked genes which were upregulated in the RNA clusters. Increasing link score correlates with gene upregulation. We also show the number of upregulated peaks for each cell type identified as cCREs. **g** Browser tracks for *Cck* with identified cCREs linked to *Cck*’s TSS. Red connections indicate a negative correlation with expression, while blue indicates a positive correlation. Example peaks (g1, g2) shown to right. **h** Peak accessibility at the TSS for *Cck* is shown both for peaks divided by cluster (top) and *Cck* expression (bottom). CCK+ cells express higher accessibility at the TSS. Differentially expressed peaks in CCK+ cells near the transcription start site are boxed in red, with one upstream of the TSS and one in the first intron of *Cck*. These overlap putative promoter-like and enhancer elements from the ENCODE repository.

We characterized the chromatin accessibility properties of PV+/CCK+ and PV+/CCK- interneurons in WT adult mouse hippocampus by analyzing snATAC-seq data obtained in our laboratory from simultaneous snRNA/ATAC profiling (10x Multiome) (*n* = 632 retained cells after TSS enrichment thresholding) (Fig. 6; Supplementary Fig. 12). The Assay for Transposase-Accessible Chromatin (ATAC) is a sequencing-based technology used to assess genome-wide chromatin accessibility. Profiling from the same nuclei from our snRNA-seq data enables integrative analysis.

Based on UMAP clustering after dimensionality reduction, we find that chromatin accessibility peaks recapitulate the PV+ interneuron cell type clusters derived from snRNA-seq analysis (Fig. 6a), and that peak quality is consistent across cell types and overlaps with known transcription start sites (TSS)(Supplementary Fig. 12a, b).

When comparing “gene activity scores”, defined by accessibility of chromatin at the TSS (a method of “inferring” gene expression from chromatin accessibility), with gene expression data, we find a strong agreement between these modalities (Fig. 6c). Differentially expressed TSS peaks (between clusters) strongly intersect differentially expressed genes (Fig. 6b). These differentially expressed genes also strongly overlap known enhancer/promoter regions (Supplementary Fig. 12c) [54]. Visual inspection of peak accessibility at the TSSs of the major marker genes which define the PV+ interneuron types show differentially expressed chromatin accessibility peaks for several cell type markers. For example, the Dock4-Eya4, Vav3, and Vipr2 cell types show increased chromatin accessibility peaks at the TSSs of the *Eya4, Vav3, and Vipr2* genes, respectively (Fig. 6d). Based on these results chromatin accessibility peak analysis largely recapitulates the PV+ interneuron types that we observed with snRNA-seq analysis and shows there are important differentially expressed chromatin accessibility peaks at the TSSs for key genes whose expression defines PV+ interneuron identity.

Identified PV+ interneuron cell types exhibit differential chromatin accessibility peaks throughout the genome (Fig. 6e, top). Similarly, PV+/CCK+ and PV+/CCK- interneurons exhibit differential chromatin accessibility peaks throughout the genome (Fig. 6e, bottom). We next identify concomitant gene expression and peak accessibility differences (peak-to-gene linkage analysis) for each gene (Supplementary Fig. 12d) [55]. To determine the effective influence of individual distal peaks (primarily corresponding to candidate enhancers) on gene expression, we compute the fraction of linked genes differentially expressed in each cell type and subtype at various linkage score thresholds. This provides a useful metric for impact assessment for candidate linkages. A linkage score of 0.1 between a cluster defined differential peak and its associated gene corresponds directly with a 20–50% probability of upregulated differential gene expression in that cluster (at a standard *p* < 0.01 for differential expression) (Fig. 6f). We then investigate differential linkages related to the *Cck* gene (Fig. 6g). In total, 15 linkages to *Cck* were identified across chromosome 9. When subdividing cells into PV+/CCK+ versus PV+/CCK- groups, peaks at all positive linkages exhibit positive log-fold changes in CCK+ compared with CCK- cells. We find the reverse pattern for negative linkages (Fig. 6g). At the *Cck* TSS (Fig. 6h), there are several striking examples of accessibility variation between clusters, as well as between PV+/CCK+ and PV+/CCK- cells. Both Vipr2 and Dock4-Chrm2 exhibit a high accessibility upregulation. Vipr2 also shows a dip in accessibility at the TSS, characteristic of transcription factor binding [56]. Chromatin accessibility peaks are increased at the *Cck* TSS in PV+/CCK+ as compared to PV+/CCK- cells. Additionally, several peaks near the TSS appear primarily in PV+/CCK+ cells (Fig. 6h). The peak upstream of the *Cck* TSS overlaps a DNase-H3k4me3 (promoter like) ENCODE region, while the downstream peak in the first intron of *Cck* overlaps an ENCODE region designated as having a proximal enhancer-like signature [57].

### Dysregulation of OXPHOS gene expression is associated with psychiatric conditions

**Fig. 7:**
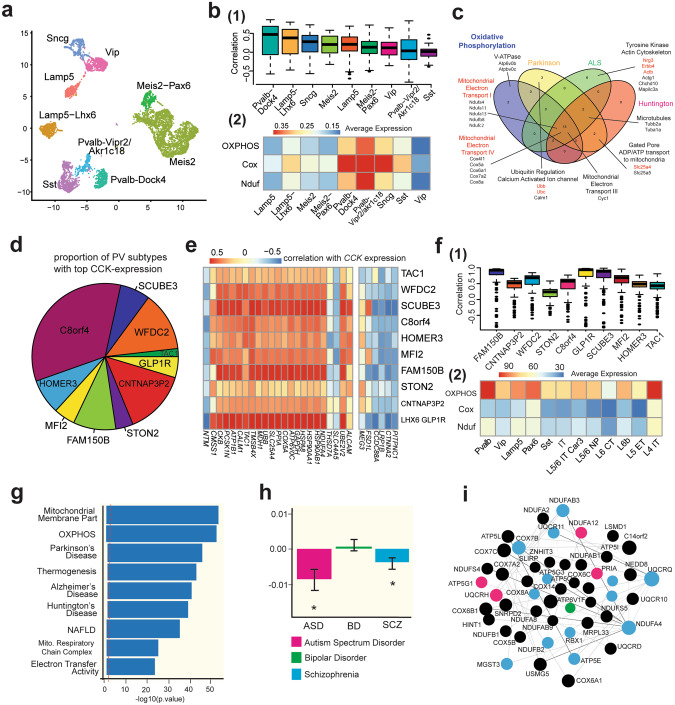
Dysregulation of OXPHOS gene expression is associated with psychiatric conditions. **a** UMAP of all inhibitory interneuron subclasses and types identified in the hippocampus. **b** (1) Boxplots of the correlation between imputed *Cck* expression, and imputed expression for all OXPHOS genes annotated to the ontology term GO:0006119 (221 genes). Inhibitory interneuron subclasses are ranked by median correlation of *Cck* and OXPHOS genes. Both Pvalb-Dock4 and Lamp5-Lhx6 show a unique co-expression of these genes. **b **(2) Mean expression of OXPHOS genes, as well as COX (mitochondrial electron transport IV) and NDUF (mitochondrial electron transport I) genes. Highest expression is in Pvalb and Sncg subclasses. Quantities thresholded to range (0, 0.35) to improve visualization. PV+ interneurons of both types exhibit significantly increased expression of all three gene types. **c** Venn diagram showing intersections of *Cck* co-expressed (see Fig. 4) genes associated to top KEGG terms (OXPHOS, Parkinson, ALS, Huntington). Schizophrenia (SCZ) and autism (ASD), neuropsychiatric disorders strongly associated with PV+ interneurons, are not directly included as a KEGG term, but have strong associations with multiple genes and structures (highlighted in red) co-expressed with *Cck*. These mechanisms include OXPHOS, as well as genes associated with the well-known ERBB pathway. **d–f** Human snRNA-seq data from Allen recapitulates our molecular characterization of mouse PV+/CCK+ interneurons in hippocampus and cortex. **d** Pie chart indicates the proportion of human cortical PV+ interneurons that contain above median imputed *CCK* expression, grouped by PV+ interneuron cell type. **e** Correlation of imputed *CCK* expression from human cortical neurons with the highly co-regulated genes identified in Fig. 4 recapitulates the positive and negative correlation results described previously in most (~88%) of genes, across nearly all PV+ interneuron types. Heatmap split by projected positive (left) and negative (right) correlation with *CCK* expression. **f** (1) Boxplot shows correlation of OXPHOS genes (187 genes) with *CCK* expression in PV+ interneuron types. Most PV+ interneuron types show strong co-expression of *CCK* with OXPHOS genes in human cortex. (2) Average expression of OXPHOS, COX, and NDUF genes within each cell type. PV+ interneurons show strong upregulation of OXPHOS and NDUF subunit genes, with slightly lower COX expression in human cortex than in mice. The latter may be influenced by four (~22%) of the COX genes showing extremely low expression (only 4.2% of OXPHOS genes experienced similar issues, including the COX genes). **g**–**i** Human cortical RNA-seq data from *Gandal et al., 2018*, reveals association of OXPHOS gene expression dysregulation with autism spectrum disorder (ASD) (*n* = 51) and schizophrenia (SCZ) (*n* = 559) compared to healthy controls (n = 936). **g** Enrichment of GO terms by gProfileR for the gene expression module. **h** Module eigengene association for each disorder with linear regression beta value displayed (FDR < 0.05). **i** Network with nodes of most representative genes within the selected module, with differential expression in each disease shown by the color of node.

Having established above that OXPHOS gene expression is highly correlated with *Cck* expression in PV+ interneurons for both mouse hippocampus and cortex, we expanded the scope of our molecular characterization of OXPHOS gene expression correlation with *Cck* expression to other interneuron subclasses and types (Fig. 7a, b). As expected based on our analyses of specific OXPHOS genes in Fig. 5, we find that OXPHOS gene expression is most positively correlated with *Cck* expression in the major PV+ interneuron types as compared to other interneuron subclasses (Fig. 7a, b). We include all annotated OXPHOS genes (GO:0006119) to avoid bias toward the identified *Cck* co-expressed genes.

In general, the correlation between OXPHOS genes and *Cck* expression is positive for all interneuron subclasses and types. We find that OXPHOS genes as well as COX/NDUF gene (mitochondrial electron transport complexes I and IV) are highly expressed in PV+ interneurons, as well as in SNCG cells to a lesser extent (Fig. 7b). Many of the OXPHOS genes that most strongly correlate with *Cck* expression in PV+ interneurons are associated with neurological disorders, identified using KEGG pathways (Top ranked terms are OXPHOS, Parkinson and ALS, *p* < 10^–10^). Though not directly included as a KEGG term, schizophrenia (SCZ) and autism spectrum disorder (ASD) have strong associations with multiple genes co-expressed with *Cck*. These mechanisms include OXPHOS, as well as genes associated to the well-known neuregulin (NRG)-ERBB pathway (Fig. 7c) [58–60], and motivated further analyses in human tissue [61–64].

We then investigated cortical post-mortem human brain tissue by analyzing snRNA-seq data available from the Allen Institute [65]. This dataset includes 47,432 cells sampled from 6 human cortical areas. 2800 PV+ interneurons are included, with types labeled as TAC1, WFDC, SCUB3, c8orf4, HOMER3, MF12, FAM150B, STON2, CNTNAP32, and LHX6-GLP1R. Approximately 27.5% of cells express at least one copy of the *CCK* gene, replicating percentages identified in mouse. After imputation using MAGIC, cells exceeding the median expression level of *CCK* are distributed across all PV+ interneuron types (Fig. 7d). We next consider those genes exhibiting co-expression with *CCK*. For each human PV+ interneuron cell type, we test the top 10% of genes co-upregulated with *Cck* that we identified previously in mouse PV+ interneurons to compare and corroborate our results across species. We find in human PV+ interneurons that most of these select genes, which include many OXPHOS genes, have expression which is strongly correlated with *CCK* expression (Fig. 7e). As expected, based on our correlation analyses in mouse, we find that OXPHOS gene expression is positively correlated with *CCK* expression in all PV+ interneuron types (Fig. 7f). To extend the scope of our inquiry even further, we assessed average expression levels of the OXPHOS gene panel in the major neuron subclasses of human cortex, including from inhibitory and excitatory neurons. With the one exception of L4 IT neurons, PV+ interneurons express the highest levels of OXPHOS genes in human cortex (Fig. 7f2).

We then interrogated a published RNA-seq data set from post-mortem human cortex obtained from 1695 individuals, including healthy controls (*n* = 936), and patients with autism spectrum disorder (ASD)(*n* = 51), schizophrenia (SCZ)(*n* = 559), or bipolar disorder (BD)(*n* = 222) [66]. To our knowledge this dataset housed in the PsychEncode repository is the largest of its kind. Based on RNA-seq data analysis we identify several gene co-expression modules, where the length of each edge of the graph is inversely proportional to the correlation of two nodes (a node is individual gene expression). One such gene co-expression module is highly enriched for gene ontology (GO) terms of “mitochondrial membrane part” and “OXPHOS” (Fig. 7g). When analyzing the eigengene for each disorder within this module it is revealed that there is a significant decrease in expression for both ASD and SCZ (FDR < 0.05) (Fig. 7h). Upon inspection of the individual genes making up this “mitochondrial membrane part” and “OXPHOS” gene expression module, it is apparent that several differentially expressed genes (*COX6A1*, *NDUF4* among others) in psychiatric patients are linked to gene-related processes that are highly enriched in PV+/CCK+ interneurons.

## Discussion

Genetic isolation of CCK+ interneurons has proven difficult as CCK is expressed in multiple subclasses of interneurons as well as excitatory neurons [29, 67–69]. By using a dual recombinase-responsive reporter line, the CCK-Cre line, and the Dlx5/6-Flp line, which expresses Flp in GABAergic neurons, we achieve specific labeling of CCK&Dlx5/6+ GABAergic inhibitory interneurons [30, 32, 41]. Our study confirms that CCK+ interneurons are abundant and heterogenous [20, 23, 32, 41, 67, 68, 70, 71]. We find that some PV+, CR+, SST+, and VIP+ cells are (CCK&Dlx5/6)+, indicating 1) that Dlx5/6+ labeling is not restricted to just the medial ganglionic eminence (MGE) OR caudal ganglionic eminence (CGE) lineages, and 2) that CCK expression findings from the earlier transcriptomic literature can be validated at the protein level in interneurons, with the expected caveat that no Cre reporter can be assumed to be absolutely faithful in all Cre-expressing cells.

Based on this we formed the hypothesis that the CCK+ interneuron phenotype may modulate distinct interneuron characteristics. We focused our investigation on PV+ interneurons because we find strong CCK expression in a subset of these cells, providing an internal control within PV+ interneurons. By generating a PV-tdTomato; CCK-Dlx5/6-GFP mouse, we determine that PV+ and CCK+ co-expressing interneurons account for 40–56% of all PV+ interneurons, and for ~1/3 of all (CCK&Dlx5/6)+ interneurons. These results from the PV-CCK co-labeled transgenic mouse corroborate our PV immunostaining and PCR results that we performed in the CCK inhibitory interneuron labeled transgenic mouse. We also find PV and CCK co-expressing interneurons in monkey, but not rat. PV/CCK co-expressing interneurons are also found in human brain [72]. We performed morphological and electrophysiological characterization of PV+/CCK+ cells and find that the somatodendritic features are similar between PV+/(CCK&Dlx5/6)+ and PV+/(CCK&Dlx5/6)- cells by our measures. Our electrophysiology experiments revealed increased afterhyperpolarization (AHP) amplitudes in PV+/CCK- interneurons compared to PV+/CCK+ interneurons. We then genetically inactivated PV+/CCK+ interneurons in hippocampal CA1 and determined behavioral performance using multiple memory tasks. We find that PV+/CCK+ interneurons in CA1 are required functionally for both the object location memory (OLM) and the fear renewal tasks.

We then determined the differences between PV+/CCK+ and PV+/CCK- interneurons in WT mice using a comprehensive multi-omic approach. We find that PV+/CCK+ interneurons are PV+ interneurons rather than CCK+ or SNCG+ interneurons [20], and that they strongly express OXPHOS genes compared to PV+/CCK- cells. This positive relationship between CCK and OXPHOS gene expression is strongest in PV+ interneurons, as compared to other major interneuron subclasses, and this is true in both hippocampus and cortex. This is biologically important as PV+ interneurons are fast-spiking and have high energy requirements to maintain this level of activity which likely also makes them susceptible to oxidative stress [5, 6, 53, 73–75].

We extend our characterization of PV+/CCK+ interneurons to human brain and find that PV+/CCK+ interneurons strongly express OXPHOS genes compared to PV+/CCK- cells, and that this positive relationship between *CCK* and OXPHOS gene expression is strongest in PV+ interneurons. PV+ interneurons may express OXPHOS genes at higher levels than almost any other major neuron subclass in human cortex that we measured (see Fig. 7f2). We then analyzed RNA-seq data from brain samples from patients with psychiatric conditions. Dysregulation of a gene module containing the OXPHOS genes is associated with both ASD and SCZ in our analysis [66]. This is important as oxidative and metabolic stress have been implicated in both ASD and SCZ [76–79].

In conclusion, our present work characterizes CCK-expressing PV+ interneurons. We demonstrate that in PV+ interneurons CCK is consistently co-expressed with OXPHOS associated genes in mouse hippocampus and cortex, and we extend these results to human brain samples. In human samples, OXPHOS genes are strongly expressed in PV+ interneurons. Finally, we demonstrate that the OXPHOS gene module is dysregulated in human brain sample from patients with an ASD or SCZ neuropsychiatric condition.

## Materials and methods

### Animals

All animals were handled, and experiments were conducted according to the NIH guidelines for animal care and use, and procedures were approved by IACUC at UCI. WT and transgenic mice of 8–12 weeks old (either sex) were used for experiments (except 4–6 weeks old for electrophysiology). The animal numbers and cell samples were calculated based on power analysis or estimation from our previous studies. The "a priori" criteria are defined before executing experiments for rational inclusion/exclusion of data, as established in our published studies. Specifically, if a technique failed (injection target was missed, for example) that data was excluded. Whenever possible, mice were randomly assigned to experimental vs. control groups with matched age and sex. Experiments were not blinded during data acquisition, but imaging and behavioral data analysis were performed blind to treatment. Please see the Supplementary Materials and Methods.

### Immunohistochemistry

For immunochemical staining experiments, animal tissue was prepared as previously described, and primary antibodies are commercially available (Supplementary Table 6). Sections were analyzed using Metamorph/Adobe Photoshop tools (see Supplementary Materials and Methods).

### Volumetric confocal and light sheet imaging

Tissues were cleared and immunostained using PEGASOS, TESOS and iDISCO protocols, and confocal and light sheet procedures were performed as previously described (see Supplementary Materials and Methods).

### Single-cell RT-PCR

The contents of fluorescent cells in hippocampal slices were extracted for reverse transcription (RT), followed by PCR for PV and CCK genes (see Supplementary Materials and Methods).

### Single-nuclei-RNA-sequencing

Nuclei were prepared from PV-Cre; cSUN1 [33] mouse hippocampus, followed by Chromium Next GEM Single Cell 3' GEM snRNA-seq library construction and sequencing on an Illumina NovaSeq 6000. Analysis was performed with Seurat v3 R (see Supplementary Materials and Methods).

### Electrophysiology and cell identification

Electrophysiology was performed on coronal brain slices from hippocampus and cell morphology confirmed GFP- and/or tdTomato‐ expressing interneurons. Imaris was used for morphometrics (see Supplementary Materials and Methods).

### 10X Multiome sequencing and data analysis

Multiomic data were generated following the 10X Genomics multiome kit guidelines for frozen brain tissue (CG000375 Rev B and CG000338 Rev E). Multiome data were processed using the combined Seurat, Signac pipeline [80, 81]. Differential expression testing of genes was accomplished using the DESeq2 statistical framework [82], with suggested additions for single-cell data. Imputation was computed using MAGIC [83]. Gene ontology/KEGG pathway analysis was computed using topGO and gProfiler2 [84, 85].

### Mouse mined data transcriptomics analysis

Raw sn/scRNA-seq was analyzed from publicly available datasets, consisting of two scRNA-seq (10x v3 and SMART-Seq) sample sets covering the isocortex and hippocampal formation, and three snRNA-seq (1 10x v2, 2 10x v3) samples taken from the primary motor cortex of the adult mouse [27]. From this 28,951 cells were labeled as PV+ interneurons (see Supplementary Materials and Methods).

### Human mined data transcriptomics analysis

Human data was obtained from the Allen institute [65]. Samples were collected from the middle temporal gyrus, anterior cingulate gyrus, primary visual cortex, primary motor cortex, primary somatosensory cortex, and primary auditory cortex. Data were analyzed using the same pipeline as 10x snRNA, but outliers, subclass labels, and cell type aliases (cell types) were retained from the Allen metadata.

Human data was also obtained from the PsychENCODE consortium integrated analysis repository [66]. Briefly, RNA-seq data from human frontal and temporal cortex were generated across eight studies, yielding 2188 samples from ASD, SCZ, BD and Controls (*n* = 1769 patients total). WGCNA was performed to identify co-expression modules using gene quantifications. Module impact was measured by their first principal component (eigengene).

### Statistical analysis

Data were presented as mean ± S.E.M. unless otherwise indicated. For statistical comparisons between groups, the data were checked for normality distribution and equal variance. If the criteria where met, a *t* test was performed to compare two groups; when the criteria were not met, a Mann-Whitney U-test was used. For statistical comparisons across more than two groups, we used One-Way ANOVA or the Kruskal–Wallis test (non-parametric One-Way ANOVA) and post hoc comparison tests for group comparisons. In all experiments, the level of statistical significance was defined as *p* < 0.05.

### Supplementary information

Supplemental Figures and Tables

Supplemental Video 1

Supplemental Video 2

Supplemental Video 3

Supplemental Video 4

Supplemental Material and Methods

## Data Availability

The data that support the findings of this study are available from the corresponding author upon reasonable request.
