# Supplementary material for: Anatomical and molecular characterization of parvalbumin-cholecystokinin co-expressing inhibitory interneurons: implications for neuropsychiatric conditions
Source: Mol Psychiatry. 2023 Jul 13;28(12):5293–308. doi: 10.1038/s41380-023-02153-5 (PMC11041731; doi:10.1038/s41380-023-02153-5)
Supplement: Supplementary file 1 — Supplemental Figures and Tables [file 41380_2023_2153_MOESM1_ESM.pdf]

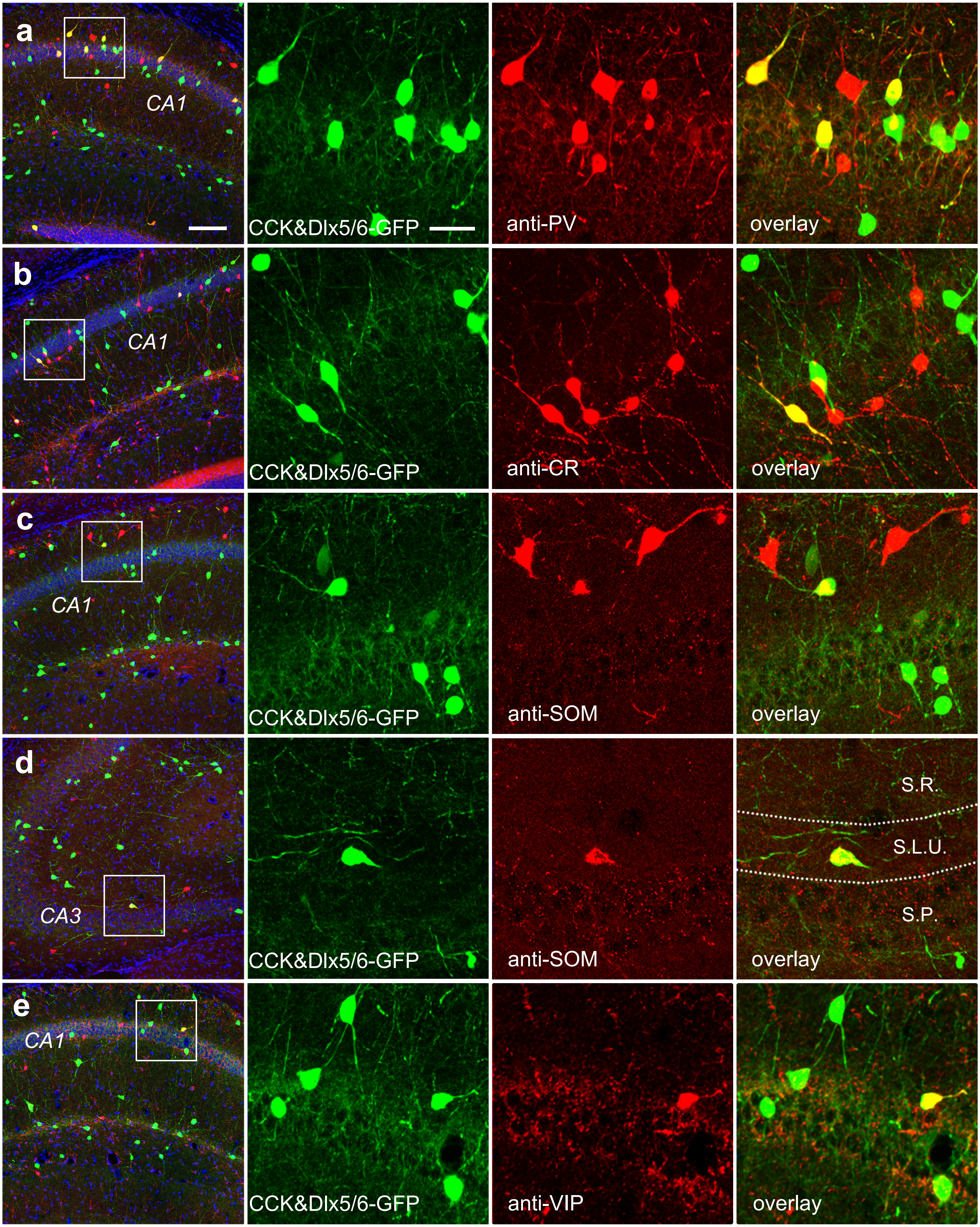

a
CA1
CCK&Dlx5/6-GFP
anti-PV
overlay
b
CA1
CCK&Dlx5/6-GFP
anti-CR
overlay
c
CA1
CCK&Dlx5/6-GFP
anti-SOM
overlay
d
CA3
CCK&Dlx5/6-GFP
anti-SOM
S.R.
S.L.U.
S.P.
overlay
e
CA1
CCK&Dlx5/6-GFP
anti-VIP
overlay

Supplemental Figure 1. Co-localization of (CCK&Dlx5/6)+ interneurons with other interneuron neurochemical marks in mouse hippocampus using immunofluorescnt antibody staining for PV, CR, SOM, and VIP. Immunofluorescent antibody staining was performed on hippocampal sections from CCK&Dlx5/6-GFP mice. (A) Hippocampal (CCK&Dlx5/6)+ interneurons (green) co-localize with PV+ interneurons (red) in both the CA1 S.O. and S.P. layers. (B) Hippocampal (CCK&Dlx5/6)+ interneurons (green) co-localize with CR+ interneurons (red) in the CA1 S.P. layer. (C) Hippocampal (CCK&Dlx5/6)+ interneurons (green) co-localize with SOM+ interneurons (red) in the CA1 S.O. layer. (D) Hippocampal (CCK&Dlx5/6)+ interneurons (green) co-localize with SOM+ interneurons (red) in the CA3 S.L.U layer. (E) Hippocampal (CCK&Dlx5/6)+ interneurons (green) co-localize with VIP+ interneurons (red) in the CA1 S.P. layer. Overlapping expression (yellow) is shown in the fourth column. The scale bar in the first panel of A applies to the first column (=100μM), while the scale bar in the second panel of A applies to second, third, and the fourth columns (=20 μM).

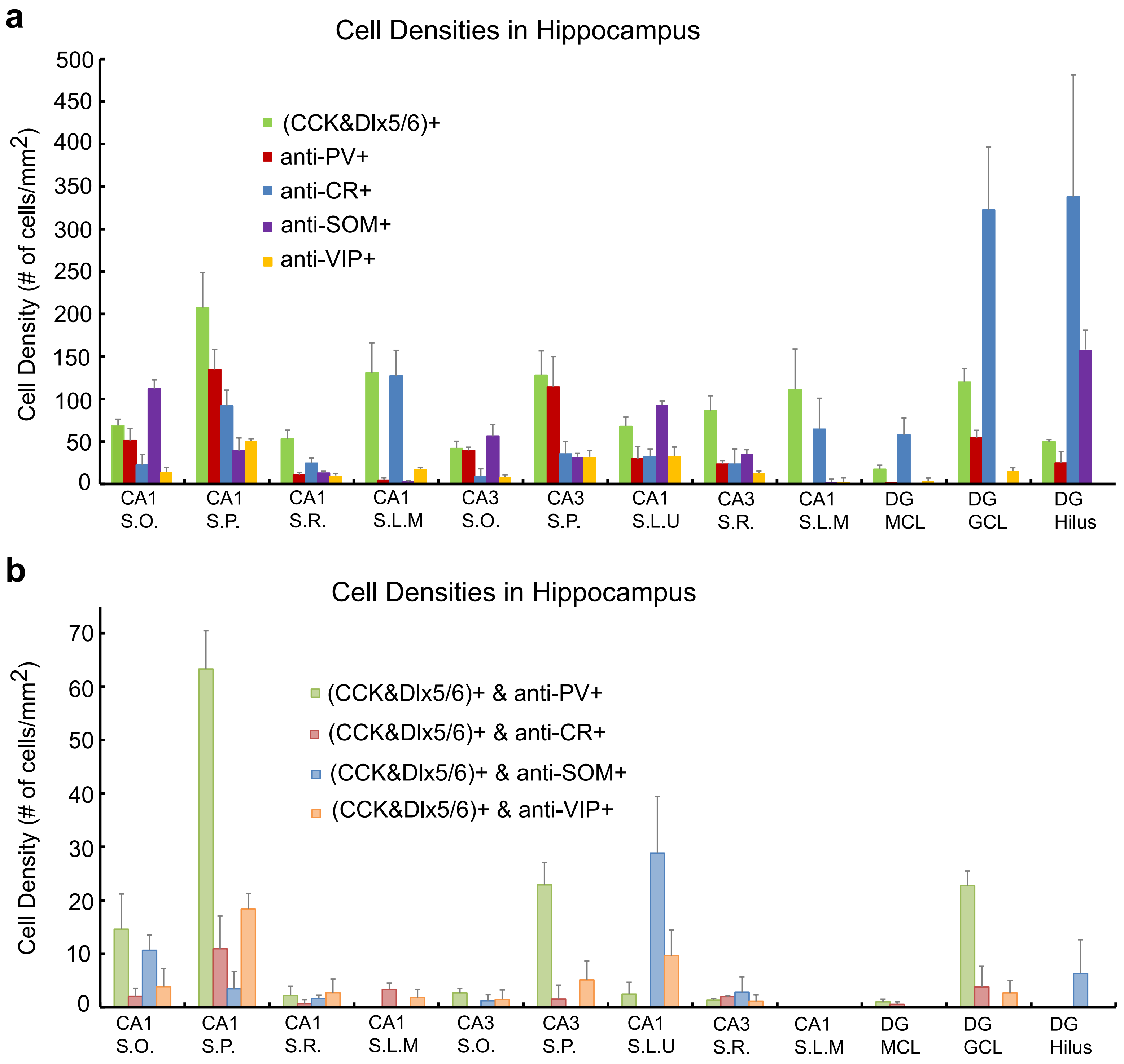
a
Cell Densities in Hippocampus
Cell Density (# of cells/mm2)
500
450
400
350
300
250
200
150
100
50
0
(CCK&Dlx5/6)+
anti-PV+
anti-CR+
anti-SOM+
anti-VIP+
CA1 S.O.
CA1 S.P.
CA1 S.R.
CA1 S.L.M
CA3 S.O.
CA3 S.P.
CA1 S.L.U
CA3 S.R.
CA1 S.L.M
DG MCL
DG GCL
DG Hilus
b
Cell Densities in Hippocampus
Cell Density (# of cells/mm2)
70
60
50
40
30
20
10
0
(CCK&Dlx5/6)+ & anti-PV+
(CCK&Dlx5/6)+ & anti-CR+
(CCK&Dlx5/6)+ & anti-SOM+
(CCK&Dlx5/6)+ & anti-VIP+
CA1 S.O.
CA1 S.P.
CA1 S.R.
CA1 S.L.M
CA3 S.O.
CA3 S.P.
CA1 S.L.U
CA3 S.R.
CA1 S.L.M
DG MCL
DG GCL
DG Hilus

Supplemental Figure 2. Quantification of the co-localization of (CCK&Dlx5/6)+ interneurons with other interneuron neurochemical marks in mouse hippocampus. Immunofluorescent antibody staining was performed on hippocampal sections from CCK&Dlx5/6-GFP mice. (A) Average densities of (CCK&Dlx5/6)+, PV+, CR+, SOM+, and VIP+ interneurons in hippocampal regions and sublamina. Note: the graph shows average densities of specific types (neurochemical maker-positive) hippocampal neurons, including PV+, CR+, SOM+, and VIP+, in specific anatomical structures of mouse hippocampus. The data is presented as mean ± SD cells/mm2 (Cell Density). CCK cell density is calculated using CCK&Dlx5/6-GFP transgenic mice (total cell counts for CCK&Dlx5/6+: n=9718 from 3 mice). PV+, CR+, SOM+, and VIP+ cell densities calculated by immunostaining (total cell counts for PV+, CR+, SOM+, and VIP+: n= 1805, 1700, 884, 383 from 3 mice, respectively). (B) Average densities of PV+/(CCK&Dlx5/6)+, CR+/(CCK&Dlx5/6)+, SOM+/(CCK&Dlx5/6)+, and VIP+/(CCK&Dlx5/6)+ cells in hippocampal regions and sublamina. Note: the graph shows average densities of neurons that co-express (CCK&Dlx5/6) and PV, (CCK&Dlx5/6) and CR, (CCK&Dlx5/6) and SOM, and (CCK&Dlx5/6) and VIP in specific anatomical structures of mouse hippocampus, respectively. The data is presented as mean ± SD cells/mm2 (Cell Density). Cell densities are calculated using CCK&Dlx5/6-GFP transgenic mice coupled with immunostaining for PV, CR, SOM, and VIP neurochemical marks (total cell counts for PV+/(CCK&Dlx5/6)+, CR+/(CCK&Dlx5/6)+, SOM+/(CCK&Dlx5/6)+, and VIP+/(CCK&Dlx5/6)+: n=516, 54, 71, and 83 from 3 mice, respectively).

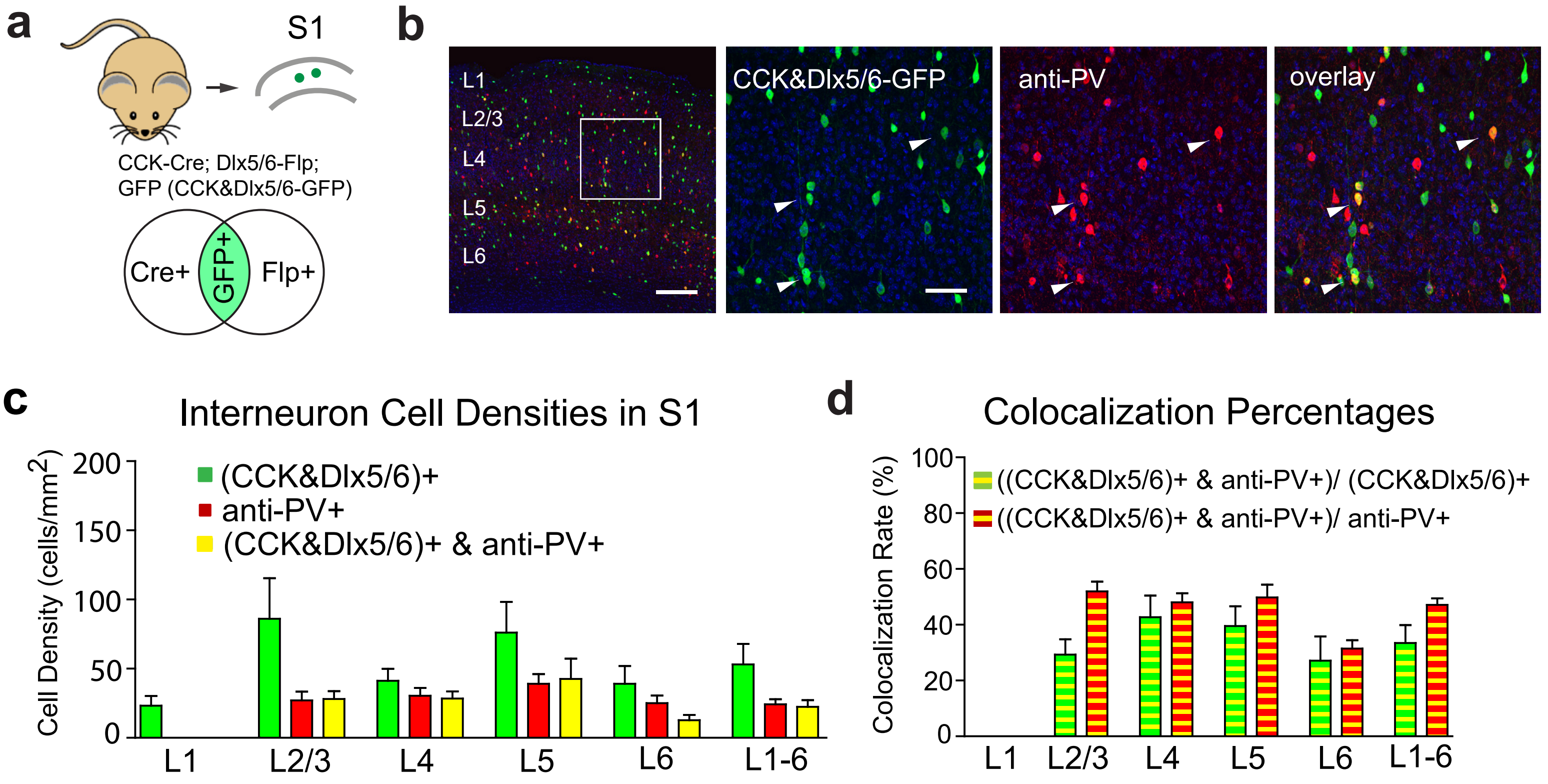

a
S1
CCK-Cre; Dlx5/6-Flp;
GFP (CCK&Dlx5/6-GFP)
Cre+
GFP+
Flp+
b
L1
L2/3
L4
L5
L6
CCK&Dlx5/6-GFP
anti-PV
overlay
c
Interneuron Cell Densities in S1
Cell Density (cells/mm2)
200
150
100
50
0
(CCK&Dlx5/6)+
anti-PV+
(CCK&Dlx5/6)+ & anti-PV+
L1
L2/3
L4
L5
L6
L1-6
d
Colocalization Percentages
Colocalization Rate (%)
100
80
60
40
20
0
((CCK&Dlx5/6)+ & anti-PV+)/ (CCK&Dlx5/6)+
((CCK&Dlx5/6)+ & anti-PV+)/ anti-PV+
L1
L2/3
L4
L5
L6
L1-6

Supplemental Figure 3. Co-localization of (CCK&Dlx5/6)+ and PV+ inhibitory interneurons in mouse cortex. (a-d) Co-localization of (CCK&Dlx5/6)+ and PV+ inhibitory interneurons in mouse somatosensory (S1) cortex using immunostaining. (a) A cartoon schematic depicting the CCK&Dlx5/6-GFP mouse line containing the CCK-ires-Cre, Dlx5/6-Flp, and RCE-dual transgenes. The Venn diagram shows that GFP expressing cells are (CCK&Dlx5/6)+ GABAergic interneurons that are Cre+ and Flp+. (b, left panel) A low magnification (4x) confocal image showing the distribution of GFP expressing neurons (green) throughout the S1 region of cortex in a coronal section from the CCK&Dlx5/6-GFP mouse. Anti-PV immunostaining signal is shown as well (red). DAPI staining shown in blue. Scale bar is 200μm. The other panels show digitally enlarged images of the white box region in the first panel. Anti-PV immunostaining (red) shows that many of the GFP-expressing cells (green) in the CCK&Dlx5/6-GFP mouse line are immunopositive for PV (yellow), as shown in the overlay in the 4th column panel. DAPI staining is shown in blue. The scale bar in the second panel applies to the second, third, and the fourth columns. The scale bar is 50 μm. (C) A histogram of the average densities (cells/mm2) of (CCK&Dlx5/6)+ (green), PV+ (red), and PV+ & (CCK&Dlx5/6)+ (yellow) interneurons in all major layers (L1, L2/3, L4, L5 and L6) of the CCK&Dlx5/6-GFP mouse S1 cortex. (CCK&Dlx5/6)+ cells outnumbered PV+ cells. (d) A histogram of the percentages of interneurons that are ((CCK&Dlx5/6)+ & anti-PV+) / anti-PV+ or ((CCK&Dlx5/6)+ & anti-PV+) / (CCK&Dlx5/6)+ in all major layers (L1, L2/3, L4, L5 and L6) of the CCK&Dlx5/6-GFP mouse S1 cortex. Counts are from 559 anti-PV+ and (CCK&Dlx5/6)+ cells, 1327 (CCK&Dlx5/6)+ only cells, and 607 anti-PV+ only cells from 6 mice.

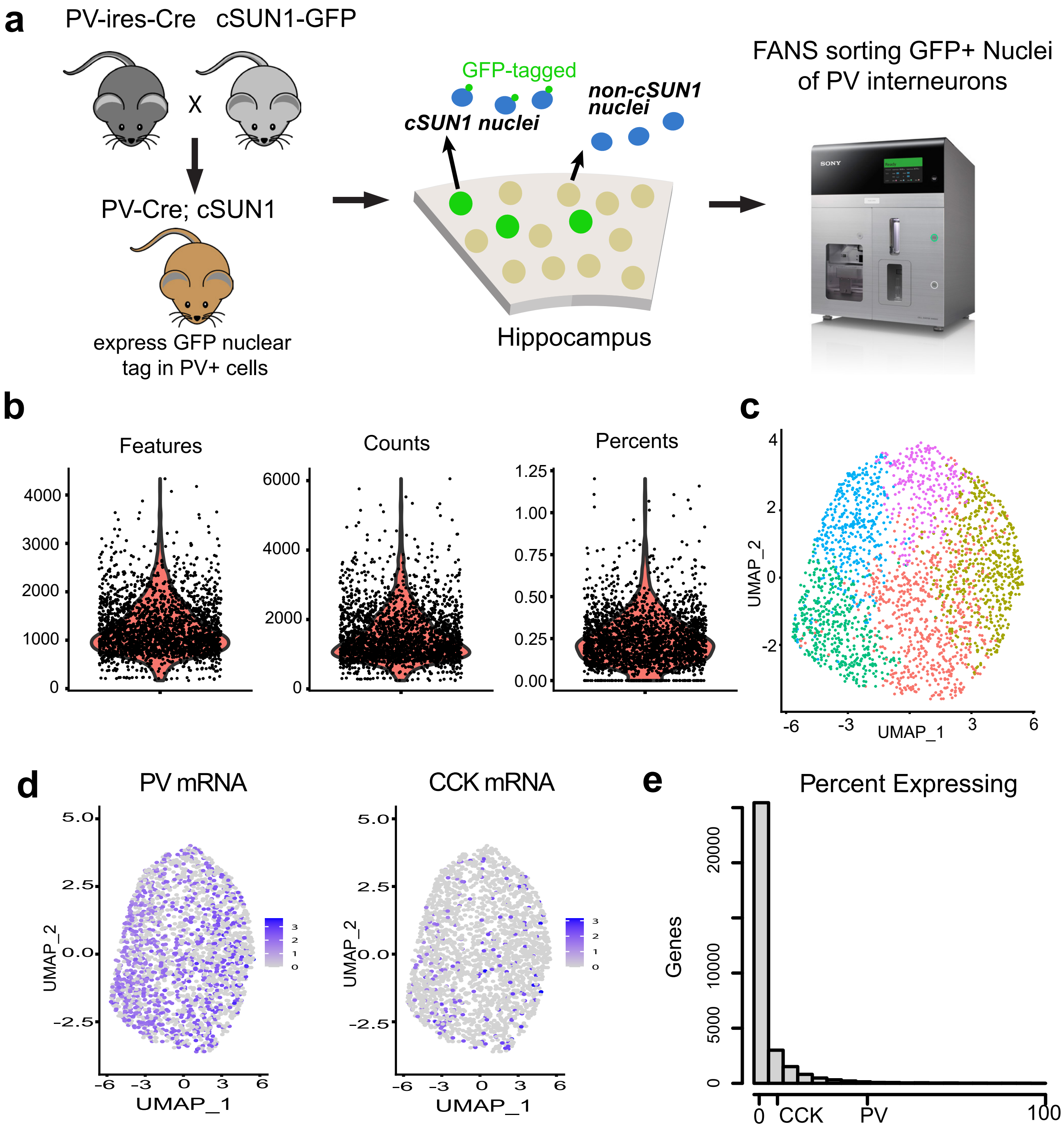
a
PV-ires-Cre
cSUN1-GFP
X
PV-Cre; cSUN1
express GFP nuclear tag in PV+ cells
GFP-tagged
cSUN1 nuclei
non-cSUN1 nuclei
Hippocampus
FANS sorting GFP+ Nuclei of PV interneurons
b
Features
Counts
Percents
c
UMAP_1
UMAP_2
d
PV mRNA
CCK mRNA
UMAP_1
UMAP_2
e
Percent Expressing
Genes
CCK
PV

Supplemental Figure 4. Co-localization of CCK+ and PV+ inhibitory interneurons in entire mouse hippocampus using sn-RNA-Seq. Analysis of sn-RNA-seq data consisting of PV expressing interneurons. Sample consisted of n=2,307 cells (after quality control). (a) PV-ires-Cre mice were crossed with cSUN1-GFP mice resulting in PV-Cre; cSUN1 progeny 30. Hippocampi from PV-Cre; cSUN1 mice were homogenized and sorted based on fluorescence (FANS) to isolate individual PV interneuron nuclei for sn-RNA-Seq. (b) Over 95% of cells have counts and features in the typical range for sn-RNA-Seq analysis. Very little data is mapped to mitochondrial DNA strands as is typical in sn-RNA-Seq. (c) UMAP projection to 2-dimensional space reveals no visually differentiated subclusters among cell groups. While Seurat did identify 5 clusters, differential testing between clusters showed the maximal average fold change of gene expression between clusters to be less than 2. (d) Raw expression levels for PV mRNA and CCK mRNA among cells projected into UMAP space. Due to dropout in sn-RNA-Seq, the gene expression matrix typically loses upwards of 96% of entries. As expected 38% of curated cells expressed PV mRNA, and roughly 8% expressed CCK mRNA. (e) Histogram demonstrates the percentage of cells expressing individual genes without imputation to account for dropout. PV mRNA is more commonly expressed than 98% of genes. CCK mRNA is more commonly expressed than 75% of genes demonstrating high expression of both PV and CCK mRNA in comparison with other genes.

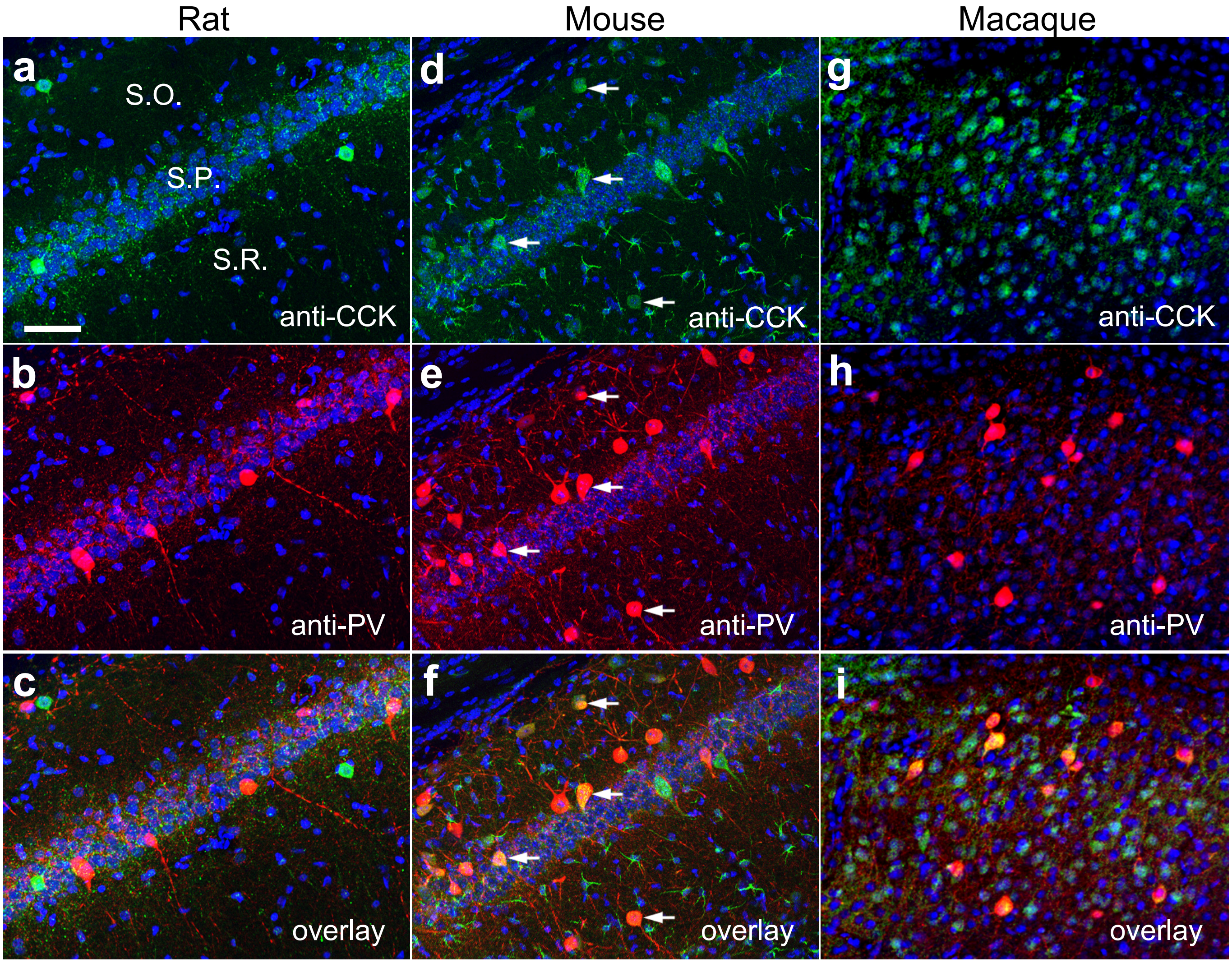

Rat
Mouse
Macaque
a
S.O.
S.P.
S.R.
anti-CCK
d
anti-CCK
g
anti-CCK
b
anti-PV
e
anti-PV
h
anti-PV
c
overlay
f
overlay
i
overlay

Supplemental Figure 5. Co-localization of CCK+ and PV+ inhibitory interneurons in mouse and macaque hippocampal sections, but not in rat hippocampal sections. Co-localization of CCK+ and PV+ inhibitory interneurons in mouse and macaque hippocampal sections, but not in rat hippocampal sections, using anti-PV and anti-CCK immunostaining. Representative coronal sections from rat, mouse and macaque hippocampus containing the CA1 region are shown. (a-c) In rat, anti-CCK immunostaining (green) and anti-PV immunostaining (red) shows no co-localiza-tion (yellow, in overlay panel) of CCK+ and PV+ interneurons in rat CA1. DAPI staining shown in blue. The scale bar is 50 μm and applies to all panels. (d-f) In mouse, anti-CCK immunostaining (green) and anti-PV immunostaining (red) shows clear co-localization (yellow, in overlay panel) of CCK+ and PV+ interneurons in CA1 SO, SP and SR sublamina. DAPI staining shown in blue. (g-i) In macaque, anti-CCK immunostaining (green) and anti-PV immunostaining (red) shows clear co-localization (yellow, in overlay panel) of CCK+ and PV+ interneurons in the CA1 SP sub-lamina (34% of PV+ interneurons are also CCK+; n=3 macaque hippocampi (138 PV+/CCK+ out of 403 PV+ interneurons). DAPI staining shown in blue.

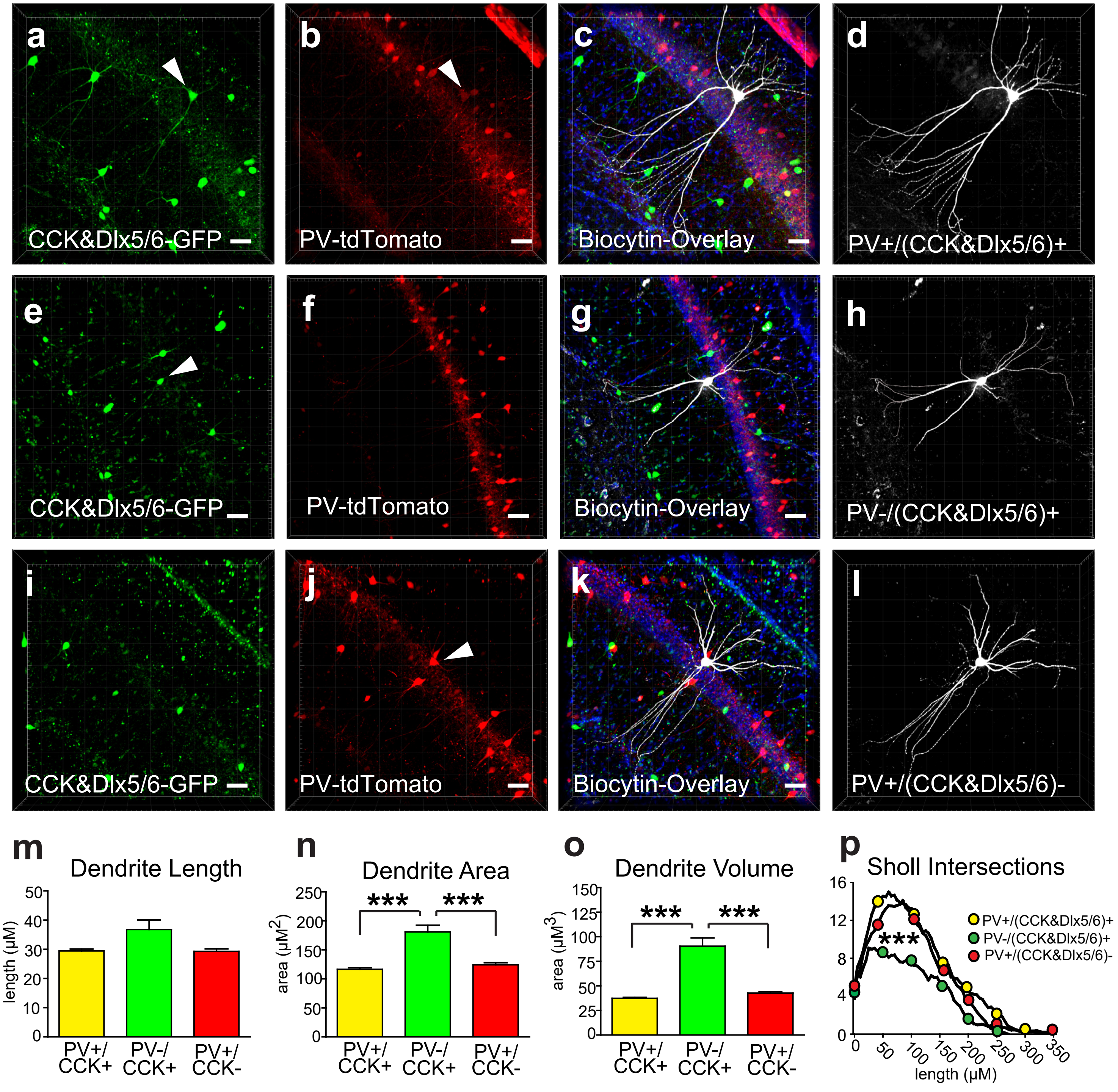

a
CCK&Dlx5/6-GFP
b
PV-tdTomato
c
Biocytin-Overlay
d
PV+/(CCK&Dlx5/6)+
e
CCK&Dlx5/6-GFP
f
PV-tdTomato
g
Biocytin-Overlay
h
PV-/(CCK&Dlx5/6)+
i
CCK&Dlx5/6-GFP
j
PV-tdTomato
k
Biocytin-Overlay
l
PV+/(CCK&Dlx5/6)-
m
Dendrite Length
length (μM)
0 10 20 30 40 50
PV+/ CCK+
PV-/ CCK+
PV+/ CCK-
n
Dendrite Area
area (μM²)
0 50 100 150 200 250
***
***
PV+/ CCK+
PV-/ CCK+
PV+/ CCK-
o
Dendrite Volume
area (μM³)
0 25 50 75 100 125 150
***
***
PV+/ CCK+
PV-/ CCK+
PV+/ CCK-
p
Sholl Intersections
0 4 8 12 16
***
PV+/(CCK&Dlx5/6)+
PV-/(CCK&Dlx5/6)+
PV+/(CCK&Dlx5/6)-
0 50 100 150 200 250 300 350
length (μM)

Supplemental Figure 6. Comparisons of morphological features of PV+/CCK+ GABA ergic inhibitory interneurons in mouse CA1. Biocytin was injected into dual-labelled cells in CA1 in ex vivo brain slices of the PV-tdTomato; CCK&Dlx5/6-GFP mouse. Sections were then imaged with confocal microscopy. Representative images are shown for PV+/(C-CK&Dlx5/6)+, PV-/(CCK&Dlx5/6)+, and PV+/(CCK&Dlx5/6)- cells. (a-d) Confocal images of a PV+/(CCK&Dlx5/6)+ GABAergic inhibitory interneuron from the PV-tdTomato; CCK&Dlx-5/6-GFP dual-labelled mouse. The cell of interest is (CCK&Dlx5/6)+ (green) and PV+ (red). The overlay panel shows co-localization of GFP and tdTomato expression (yellow). Arrow-heads point to the dual-labelled cell. Biocytin fill of the cell is shown with the other channels or by itself for morphological clarity. (e-h) Confocal images of a PV-/(CCK&Dlx5/6)+ GABAer-gic inhibitory interneuron from the PV-tdTomato; CCK&Dlx5/6-GFP dual-labelled mouse. The cell of interest is (CCK&Dlx5/6)+ (green) and PV- (red). Arrowhead points to the PV-/(C-CK&Dlx5/6)+ cell. (i-l) Confocal images of a PV+/(CCK&Dlx5/6)- GABAergic inhibitory inter-neuron. The cell of interest is (CCK&Dlx5/6)- (green) and PV+ (red). Arrowhead points to the PV+/(CCK&Dlx5/6)- labelled cell. Scale bar is 50μm for all panels. (m-p) Morphological anal-ysis of cells using the Imaris software. (m) Average dendritic length was not significantly different between PV+/(CCK&Dlx5/6)+ (n=22), PV-/(CCK&Dlx5/6)+ (n=9), and PV+/(C-CK&Dlx5/6)- (n=14) cells. (n) The avergage dendritic area was significantly increased in PV-/CCK&Dlx5/6+ cells as compared to PV+/CCK&Dlx5/6+ or PV+/(CCK&Dlx5/6)- cells. Similarly, the average dendritic volume was significantly increased in PV-/(CCK&Dlx5/6)+ cells as compared to PV+/(CCK&Dlx5/6)+ or PV+/(CCK&Dlx5/6)- cells. Sholl analysis of dendritic branching with distance along the dendrite shows that PV-/(CCK&Dlx5/6)+ cells had significantly less dendritic branching along the dendrite as compared to PV+/(CCK&Dlx-5/6)+ or PV+/(CCK&Dlx5/6)- cells. Data are presented as the mean ± S.E.M. *$P < 0.05$.

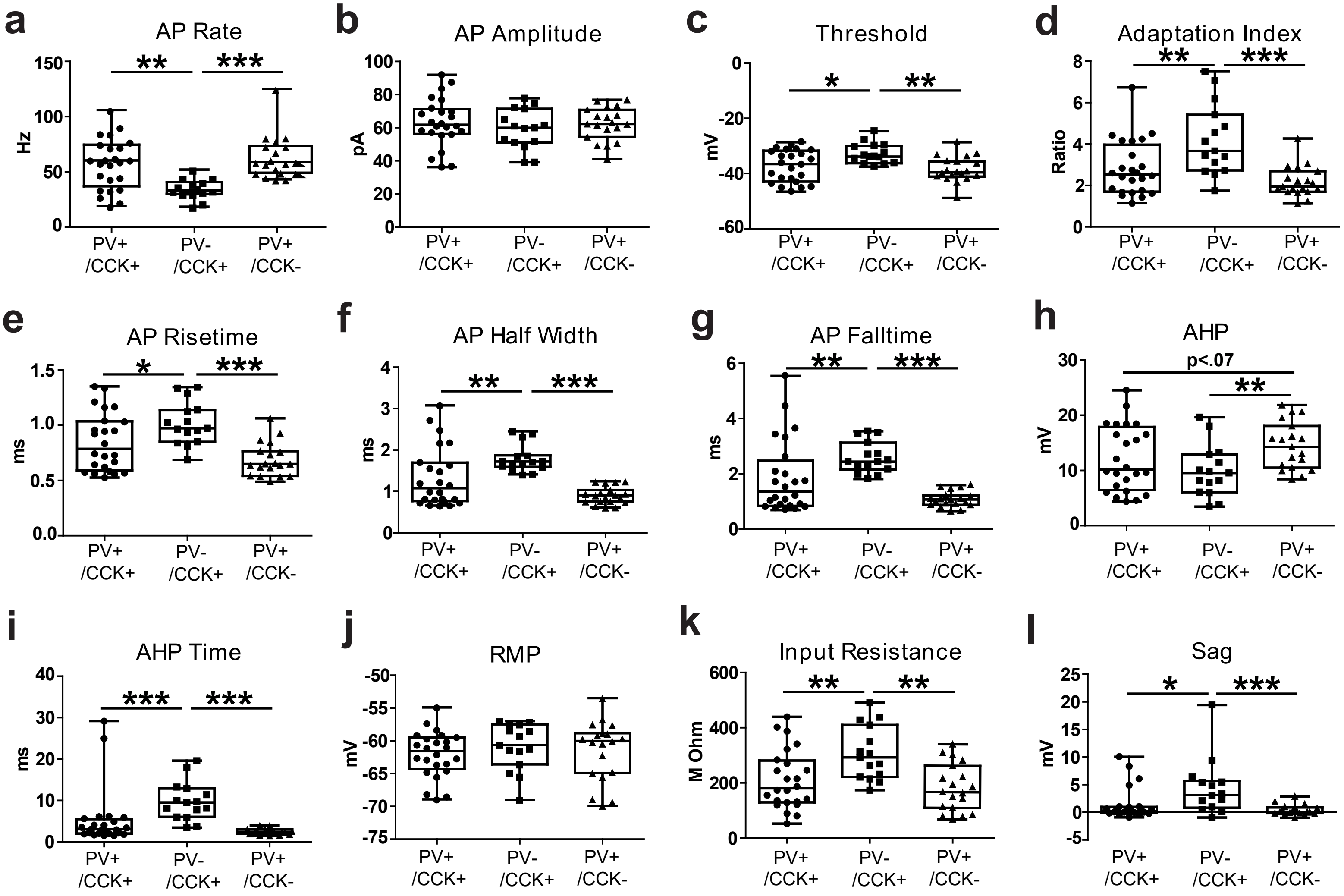

a
AP Rate
Hz
**
***
b
AP Amplitude
pA
c
Threshold
mV
*
**
d
Adaptation Index
Ratio
**
***
e
AP Risetime
ms
*
***
f
AP Half Width
ms
**
***
g
AP Falltime
ms
**
***
h
AHP
mV
p<.07
**
i
AHP Time
ms
***
***
j
RMP
mV
k
Input Resistance
M Ohm
**
**
l
Sag
mV
*
***
PV+ /CCK+
PV- /CCK+
PV+ /CCK-

Supplemental Figure 7. Comparisons of firing patterns and spike shapes of PV+/CCK+, GABAergic inhibitory interneurons in mouse CA1. Summary graphs of electrophysiological properties including, action potential (AP) frequency, AP amplitude, AP threshold, AP adaptation index, AP risetime, AP half width, AP falltime, after hyperpolarization potential (AHP), AHP time, resting membrane potential (RMP), input resistance and hyperpolarization current (-100pA) induced inward rectification “sag” from PV+/(CCK&Dlx5/6)+ (n=24), PV-/(CCK&Dlx5/6)+ (n=15) and PV+/(CCK&Dlx5/6)- (n=19) interneurons. The boxes are the 25th and 75th percentiles of the data with the median line in the middle. The whiskers are the minima and maxima. The dots represent single data points (PV+/(CCK&Dlx5/6)+ are circles, PV-/(CCK&Dlx5/6)+ are squares, and PV+/(CCK&Dlx5/6)- are triangles). PV+/(CCK&Dlx5/6)+ interneurons are similar to PV+/(CCK&Dlx5/6)- interneurons.

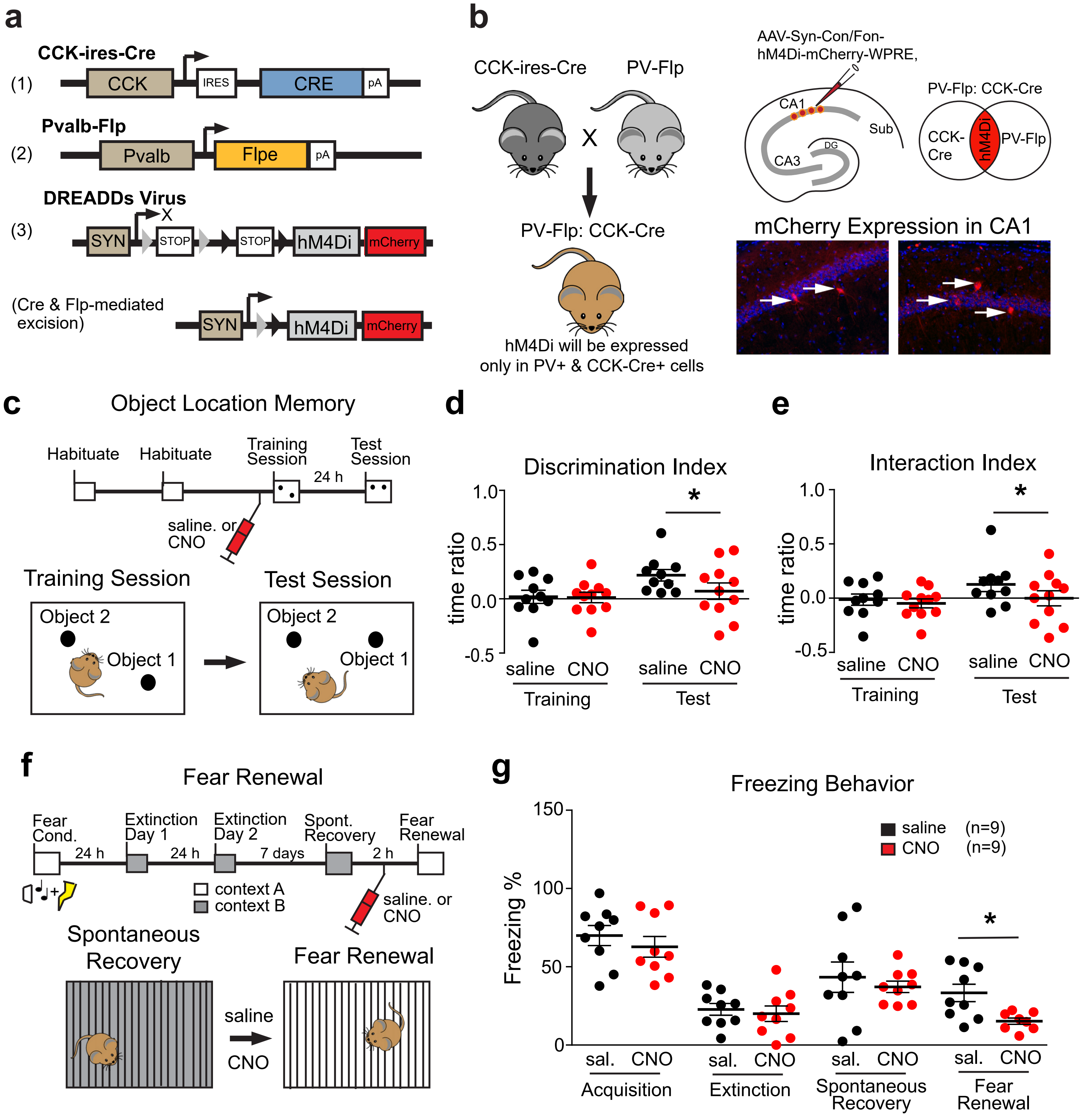
a
CCK-ires-Cre
(1)
CCK
IRES
CRE
pA
Pvalb-Flp
(2)
Pvalb
Flpe
pA
DREADDs Virus
(3)
SYN
STOP
STOP
hM4Di
mCherry
X
(Cre & Flp-mediated excision)
SYN
hM4Di
mCherry
b
CCK-ires-Cre
PV-Flp
X
PV-Flp: CCK-Cre
hM4Di will be expressed only in PV+ & CCK-Cre+ cells
AAV-Syn-Con/Fon-hM4Di-mCherry-WPRE,
CA1
Sub
DG
CA3
PV-Flp: CCK-Cre
CCK-Cre
hM4Di
PV-Flp
mCherry Expression in CA1
c
Object Location Memory
Habituate
Habituate
Training Session
Test Session
24 h
saline. or CNO
Training Session
Test Session
Object 2
Object 1
Object 2
Object 1
d
Discrimination Index
time ratio
1.0
0.5
0.0
-0.5
*
saline
CNO
saline
CNO
Training
Test
e
Interaction Index
time ratio
1.0
0.5
0.0
-0.5
*
saline
CNO
saline
CNO
Training
Test
f
Fear Renewal
Fear Cond.
Extinction Day 1
Extinction Day 2
Spont. Recovery
Fear Renewal
24 h
24 h
7 days
2 h
context A
context B
saline. or CNO
Spontaneous Recovery
Fear Renewal
saline
CNO
g
Freezing Behavior
Freezing %
150
100
50
0
saline (n=9)
CNO (n=9)
*
sal.
CNO
sal.
CNO
sal.
CNO
sal.
CNO
Acquisition
Extinction
Spontaneous Recovery
Fear Renewal

Supplemental Figure 8. Genetically targeted inactivation of PV+/CCK+ GABAergic inhibitory interneurons in mouse CA1 impairs cognitive memory behaviors. (a) Schematic illustration of genetic inactivation of PV+/CCK+ interneurons using a cross between CCK-IRES-Cre mice and PV-Flp mice (PV-Flp; CCK-Cre). (b) The resulting progeny are injected with AAV-Syn-Con/-Fon-hM4Di-mCherry-WPRE into CA1. Histological analysis in coronal brain sections verifies spatially restricted hM4Di-mCherry expression in CA1. Scale bars is 50μm. The viral injection experiment was independently repeated in 18 mice, with similar results obtained each time. (c) Scheme for the experimental design of the object-location memory (OLM) task with hM4Di/C-NO-mediated inhibition of PV+/CCK+ interneurons in CA1 during the learning phase. The box represents the open-field arena, and the black filled circles indicate the training (left) and test (right) object locations. Before the experiment, mice were handled and habituated to the con-text in the absence of objects. Mice received a single intraperitoneal injection of control saline or experimental CNO treatment (1.4 mg per kg) 1 hour before the learning session. (d) The discrimination index (DI) for the testing session 24 h after training. CNO-treated mice did not show a preference for the moved object in contrast to saline-treated controls. Data are present-ed as the mean ± S.E.M. $^*p < 0.05$ (Mann-Whitney test). (e) The interaction index for the testing session 24 h after training. CNO-treated mice did not show a preference for the moved object in contrast to saline-treated controls. Data are presented as the mean ± S.E.M. $^*p < 0.05$ (Mann-Whitney test). (f) Scheme for the experimental design of the fear renewal paradigm with hM4Di/CNO-mediated inhibition of PV+/CCK+ interneurons in CA1 during the fear renewal session. The boxes represent the shock box contexts, and the vertical lines indicate the foot shock bars. Mice received a single intraperitoneal injection of control saline (n=10) or experi-mental CNO treatment (1.4 mg per kg)(n=10) 1 hour before the fear renewal session. (g) Freez-ing behavior across all sessions. Mice first acquired fear conditioning by receiving paired sound and foot shock in the first context. The following 2 days, fear extinction sessions were performed in another context where sound was present, but without foot shock. One week later spontaneous recovery and fear renewal were measured. Fear renewal was significantly reduced in PV-Flp; CCK-Cre treated with CNO (n=9) 1 hour after the spontaneous recovery session as compared to transgenic mice treated with saline (n=9). Data are presented as the mean ± S.E.M. $^*p < 0.05$ (two-tailed t-test). PV-Flp; CCK-Cre mice performed normally in all sessions. In G, data for CNO-treated mice is in red and saline is shown in black as separate groups, although treatments were not performed until after the spontaneous recovery session.

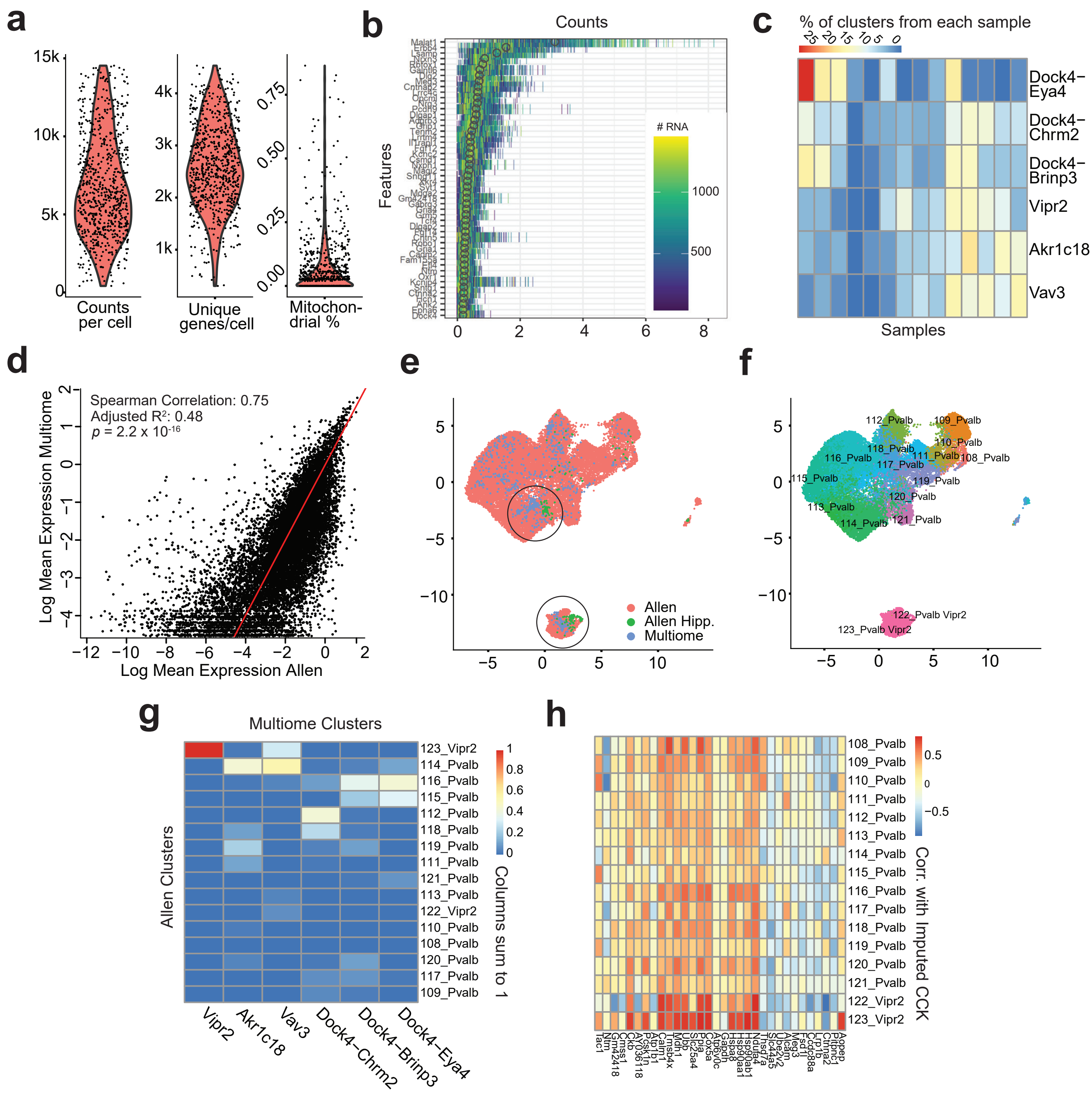
a
Counts per cell
Unique genes/cell
Mitochon-drial %
b
Counts
Features
RNA
c
% of clusters from each sample
Dock4−Eya4
Dock4−Chrm2
Dock4−Brinp3
Vipr2
Akr1c18
Vav3
Samples
d
Spearman Correlation: 0.75
Adjusted R²: 0.48
p = 2.2 x 10-16
Log Mean Expression Multiome
Log Mean Expression Allen
e
Allen
Allen Hipp.
Multiome
f
g
Multiome Clusters
Allen Clusters
Columns sum to 1
h
Corr. with Imputed CCK

Supplemental Figure 9. Characteristis of our multiome hippocampal data set, and comparison with hippocampal transcriptomic data from the Allen Institute. (a) Quality control metrics for Multiome hippocampal data. Thresholds for mitochondrial reads were set to 1%, and at least 200 unique genes were required per cell. (b) Number of counts per gene across cells for the highest expressing genes in the dataset. (c) Number of cells per sample (x-axis) in each cluster (y-axis). (c) Correlation between Multiome hippocampal data and the Allen Institute snRNA cortex and hippocampus 10X dataset. Regression line computed using unweighted linear regression. (e) Integration of Multiome hippocampal and Allen datasets using Harmony reveals distinct differences between Vipr2+ (bottom circled region) and remaining PV+ interneurons. Several regions exhibit strong representations of both our Multiome hippocampal snRNA cells and hippocampal snRNA cells from the Allen dataset (circled regions). (f) Inherited labels from the Allen snRNA-seq clustering. (g) Percentage of each Multiome cluster (x-axis) annotated to Allen clusters (y-axis) via K-nearest neighbors on Harmony integrated PCA embeddings. (h) Correlation between imputed CCK expression and gene expression replicates primary results in both cortical and hippocampal enriched clusters.

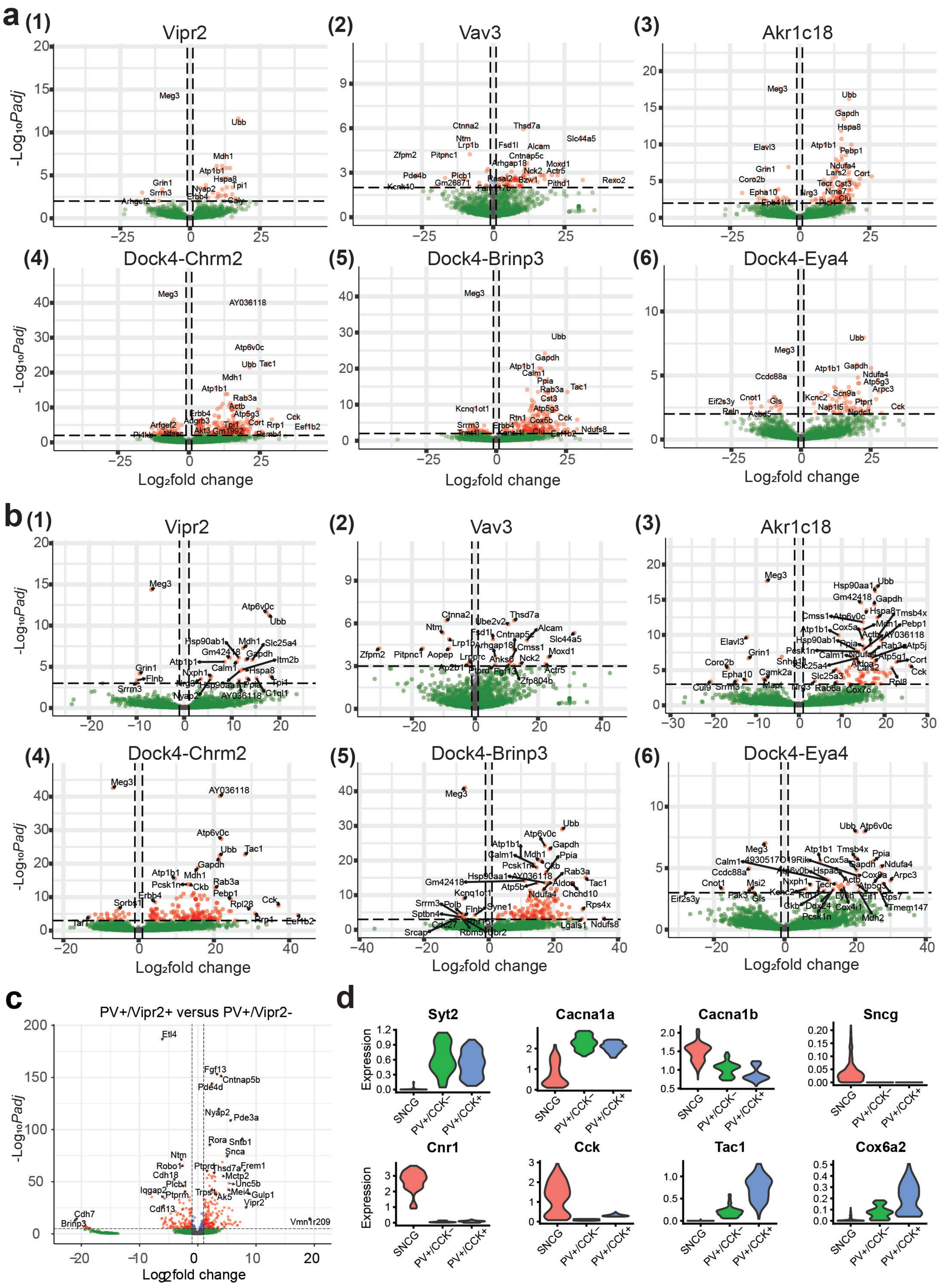
a
(1) Vipr2
(2) Vav3
(3) Akr1c18
(4) Dock4-Chrm2
(5) Dock4-Brinp3
(6) Dock4-Eya4
-Log10Padj
Log2fold change
Meg3
Ubb
Mdh1
Atp1b1
Hspa8
Tpi1
Nyap2
Erbb4
Grin1
Srrm3
Arhgef2
Caly
Ctnna2
Thsd7a
Slc44a5
Ntm
Lrp1b
Fsd1l
Alcam
Zfpm2
Pitpnc1
Cntnap5c
Arhgap18
Moxd1
Nck2
Actr5
Pde4b
Plcb1
Rasal2
Bzw1
Gm26871
Pithd1
Rexo2
Kcnk10
Gapdh
Elavl3
Pebp1
Ndufa4
Lars2
Cort
Coro2b
Tecr
Cst3
Epha10
Nrg3
Nme7
Clu
Plcl4
AY036118
Atp6v0c
Tac1
Rab3a
Actb
Atp5g3
Cck
Cort
Rrp1
Eef1b2
Arfgef2
Adgrb3
Tpi1
Pi4kb
Nfasc
Akt3
Gm1992
Psmb4
Calm1
Ppia
Cst3
Kcnq1ot1
Rtn1
Cox5b
Srrm3
Erbb4
Trmt1l
Ndufs8
Scn9a
Ccdc88a
Eif2s3y
Cnot1
Gls
Kcnc2
Ptprt
Arpc3
Reln
Acsl5
Nap1l5
Npdc1
b
(1) Vipr2
(2) Vav3
(3) Akr1c18
(4) Dock4-Chrm2
(5) Dock4-Brinp3
(6) Dock4-Eya4
-Log10Padj
Log2fold change
Meg3
Atp6v0c
Ubb
Hsp90ab1
Mdh1
Slc25a4
Gm42418
Gapdh
Itm2b
Atp1b1
Calm1
Hspa8
Grin1
Nxph1
Flnb
Nrg3
Tpi1
Hsp90aa1
Ppia
Srrm3
Nyap2
AY036118
C1ql1
Ctnna2
Ube2v2
Thsd7a
Alcam
Fsd1l
Cntnap5
Slc44a5
Lrp1b
Arhgap18
Cmss1
Moxd1
Zfpm2
Pitpnc1
Aopep
Lrrc
Anks6
Nck2
Ap2b1
Ptprd
Fgf13
Actr5
Zfp804b
Hsp90aa1
Tmsb4x
Cox5a
Pebp1
Actb
Rab3a
Atp5j
Elavl3
Pcsk1n
Snhg11
Atp5g1
Cort
Coro2b
Slc25a4
Lars2
Cck
Epha10
Camk2a
Slc25a3
Cul9
Mapt
Rab6a
Cox7c
Rpl8
Sorbs1
Erbb4
Ckb
Tarf
Rpl28
Rrp1
Eef1b2
Chchd10
Aldoa
Ndufa4
Rps4x
Lgals1
Ndufs8
Polb
Sptbn4
Cdc27
Srcap
Syne1
Ubr2
Rbm5
4930517O19Rik
Ccdc88a
Msi2
Pak3
Kcnc2
Lypd1
Eif1
Cox4i1
Pcsk1n
Ddx24
Mdh2
Tmem147
Rps7
Arpc3
Cox8a
c
PV+/Vipr2+ versus PV+/Vipr2-
-Log10Padj
Log2fold change
Etl4
Fgf13
Cntnap5b
Pde4d
Nyap2
Pde3a
Rora
Snfb1
Snca
Ntm
Robo1
Ptprd
Thsd7a
Frem1
Cdh18
Mctp2
Unc5b
Plcb1
Iqgap2
Ptprm
Trps1
Mei4
Gulp1
Ak5
Vipr2
Cdh13
Cdh7
Brinp3
Vmn1r209
d
Syt2
Cacna1a
Cacna1b
Sncg
Cnr1
Cck
Tac1
Cox6a2
Expression
SNCG
PV+/CCK−
PV+/CCK+

Supplemental Figure 10. Comparative gene expression in PV+/CCK+ and PV+/CCK- hippocam-pal neurons for each identified PV+ interneuron type. (a1-a6) Individual volcano plots for CCK+ (right side upregulated) vs CCK- (left side upregulated) cells defined by detection of at least one RNA gene. (b1-b6) Individual volcano plots treating imputed *Cck* expression as a continuous variable. One unit increase in the x direction translates to the expected expression increase associated to a unit increase in imputed *Cck* expression. (C) Volcano plots based on *Cck* expression: (1) gene expression differences in *Vipr2* expressing cells versus *Vipr2* negative cells. (d) Gene expression of typical CCK+ or PV+ interneuron function exhibited significant differences between PV+ and PV- (SNCG+) group-ings, as would be expected from their disparate lineages. Additionally, *Cck*, *Tac1* and *Cox6a2* exhibit large differences between PV+/CCK+ and PV+/CCK- groupings. Data are presented as violin plots.

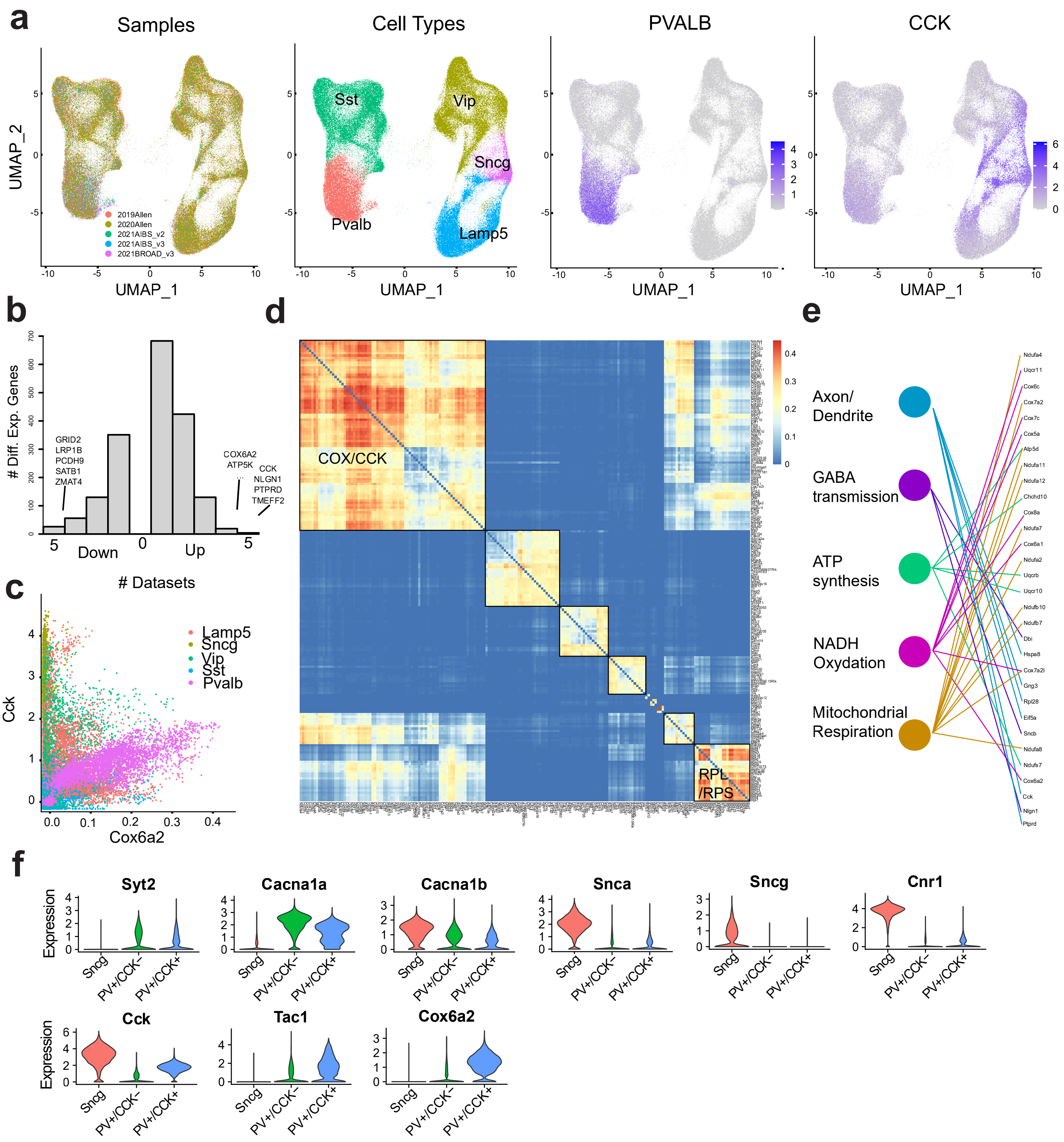

a
Samples
Cell Types
PVALB
CCK
UMAP_2
UMAP_1
2019Allen
2020Allen
2021AIBS_v2
2021AIBS_v3
2021BROAD_v3
Sst
Vip
Sncg
Pvalb
Lamp5
b
Diff. Exp. Genes
GRID2
LRP1B
PCDH9
SATB1
ZMAT4
COX6A2
ATP5K
CCK
NLGN1
PTPRD
TMEFF2
Down
Up
c
Datasets
Cck
Cox6a2
Lamp5
Sncg
Vip
Sst
Pvalb
d
COX/CCK
RPL
/RPS
e
Axon/
Dendrite
GABA
transmission
ATP
synthesis
NADH
Oxydation
Mitochondrial
Respiration
f
Syt2
Cacna1a
Cacna1b
Snca
Sncg
Cnr1
Cck
Tac1
Cox6a2
Expression
Sncg
PV+/CCK−
PV+/CCK+

Supplemental Figure 11. Comparisons of transcriptomes of PV+/CCK+ and PV+/CCK- GABAergic inhibitory interneurons in mouse. (a) (Samples) Individual sn-RNA-Seq datasets were processed as follows. Cells exhibiting an extremely high or low number of features were eliminated. Count matrices were log-normalized and scaled. Highly variable genes were identified and used as features for PCA dimensionality reduction. Next, anchors were identified and datasets were integrated via the Seurat framework. The data was projected to two dimensions via UMAP for visualization. (Cell Types) The integrated datasets were clustered, and cell types were identified via expression of interneuron gene markers (Sst, Pvalb, Vip, Lamp5, and Sncg). These are characteristically divided into the MGE (SST, PV) and CGE (VIP, LAMP5, and SNCG) lineages. (Pvalb) *Pvalb* gene expression, imputed using MAGIC, shows strong localization of *Pvalb* expression to the PV subtype. (Cck) *Cck* gene expression, imputed using MAGIC, is exhibited in both SNCG and PV interneurons. (b) Individual datasets are further analyzed through the following pipeline. *Cck* expression is imputed using MAGIC, and PV interneurons were identified as PV+/CCK+ if *Cck* expression is above the 75th percentile, and PV+/CCK- if *Cck* expression is below the 25th percentile. Differential expression testing between the groups identified >50 genes differentially expressed in at least four datasets. Many of these genes were associated to differences in interneuron communication (Cck, Nlgn1, Ptprd, Cox6a2, Grid2, Pcdh9). (c) *Cck* and *Cox6a2* expression (post imputation) are highly correlated in the PV+ cell type (0.73), but not in any other interneuron subtype. (d) Gene co-expression analysis via WGCNA identified several highly coexpressed modules. Genes exhibiting coexpression less than 0.15 were removed from the visualization. The rest clustered into 6 groupings, including a COX grouping, that was also highly co-expressed with CCK. (e) Differentially expressed genes and genes in the COX/CCK module were analyzed for their associated ontologies. Overexpressed ontologies included Axon/Dendrite, Regulation of Synaptic Transmission, Mitochondrial ATP synthesis, Electron Transfer, and Mitochondrial respiration. These ontologies were associated to mostly non-overlapping gene groupings, possibly indicating largely independent functional subclusters within the COX/CCK module. (f) Gene expression of typical CCK+ or PV+ interneuron function exhibited significant differences between PV+ and PV- (SNCG+) groupings, as would be expected from their disparate lineages. Additionally, *Cck, Tac1 and Cox6a2* expression exhibit large differences between PV+/CCK+ and PV+/CCK- groupings. Data are presented as violin plots.

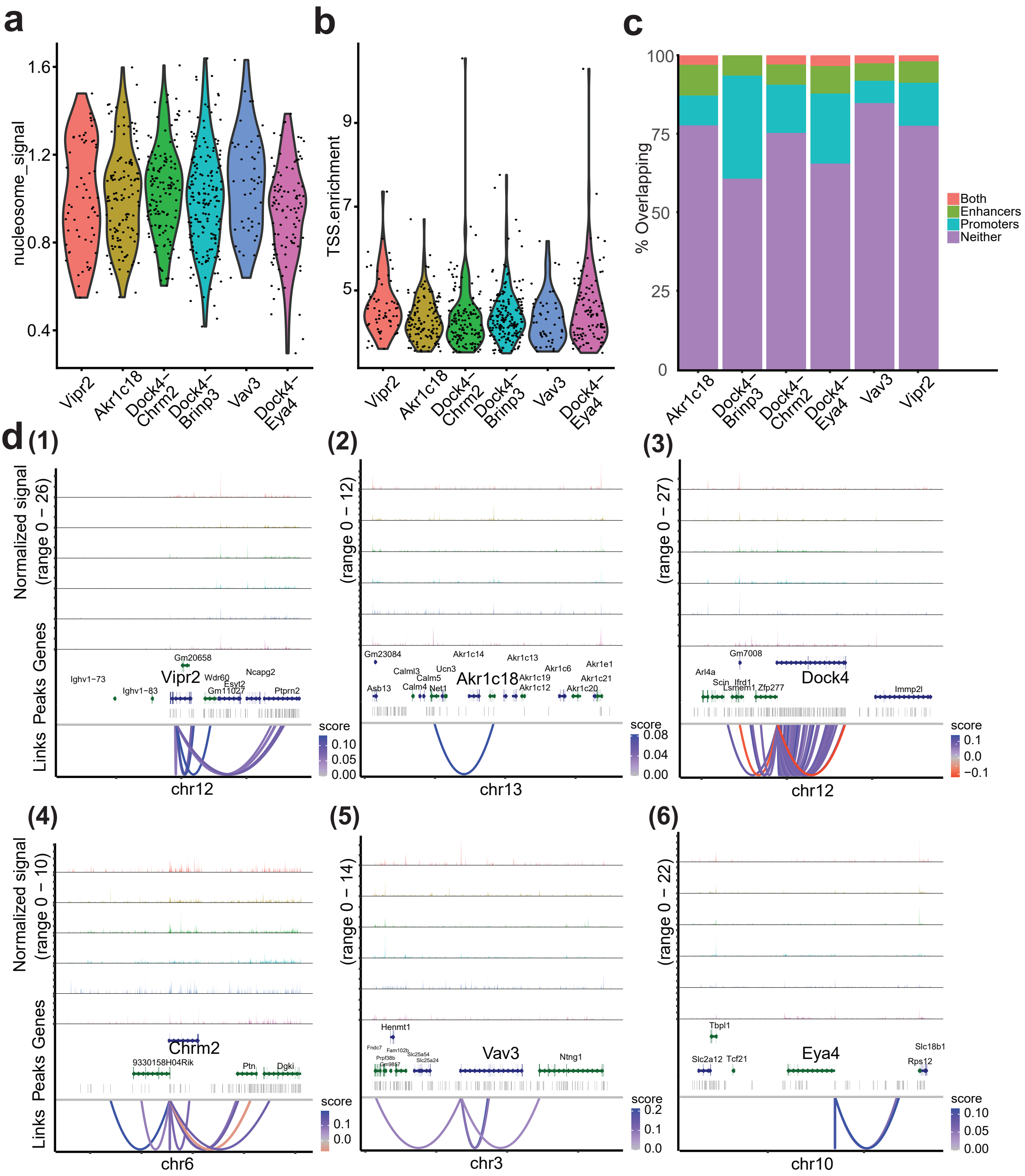

a
nucleosome_signal
1.6
1.2
0.8
0.4
Vipr2
Akr1c18
Dock4-Chrm2
Dock4-Brinp3
Vav3
Dock4-Eya4
b
TSS.enrichment
9
7
5
c
% Overlapping
100
75
50
25
0
Both
Enhancers
Promoters
Neither
d
(1)
Normalized signal (range 0 – 26)
Genes
Peaks
Links
Vipr2
Gm20658
Ighv1-73
Ighv1-83
Wdr60
Esyt2
Gm11027
Ncapg2
Ptprn2
score
0.10
0.05
0.00
chr12
(2)
(range 0 – 12)
Gm23084
Asb13
Calml3
Calm5
Calm4
Net1
Ucn3
Akr1c14
Akr1c18
Akr1c13
Akr1c19
Akr1c12
Akr1c6
Akr1e1
Akr1c21
Akr1c20
score
0.08
0.04
0.00
chr13
(3)
(range 0 – 27)
Gm7008
Arl4a
Scin
Ifrd1
Lsmem1
Zfp277
Dock4
Immp2l
score
0.1
0.0
−0.1
chr12
(4)
Normalized signal (range 0 – 10)
Chrm2
9330158H04Rik
Ptn
Dgki
score
0.1
0.0
chr6
(5)
(range 0 – 14)
Henmt1
Fndc7
Prpf38b
Fam102b
Gm9857
Slc25a54
Slc25a24
Vav3
Ntng1
score
0.2
0.1
0.0
chr3
(6)
(range 0 – 22)
Tbpl1
Slc2a12
Tcf21
Eya4
Slc18b1
Rps12
score
0.10
0.05
0.00
chr10

Supplemental Figure 12. Characteristics of chromatin accessibility peaks in hippocampal PV+ interneurons. (a-b) Nucleosome signal and TSS enrichment scores for Multiome samples. TSS enrichment was thresholded at 3.5 (in our experience Signac produces lower average TSS scores compared with alternative methods such as ArchR and SnapATAC). (c) Percentage of differentially expressed peaks overlapping known enhancer and promoter regions, divided by cluster. (d) Peak to gene linkages for each cluster marker (excluding Brinp3 as none were identified), along with coverage tracks for each cluster. Only nearby (within 1,000,000 bp of annotated gene center) candidate linkages are shown.

Supplemental Table 1. Characterization of GFP-expressing neurons in CCK&Dlx5/6-GFP transgenic mice

CCK&Dlx5/6-GFP mice		% CCK Positive	% GABA Positive
CA1	S.O.	95.4± 2.3	95.5± 1.6
	S.P.	98.0± 1.3	94.1± 1.4
	S.R.	97.3± 3.0	96.2± 0.4
	S.L.M	94.7± 1.2	96.9± 0.7
CA3	S.O.	95.5± 0.2	98.1± 1.6
	S.P.	95.8± 2.9	93.8± 1.7
	S.L.U.	97.7± 2.4	96.5± 0.9
	S.R.	98.0± 1.8	98.4± 1.6
	S.L.M	100.0	100.0
DG	ML	100.0	99.5± 0.9
	GCL	97.3± 0.8	94.8± 2.1
	Hilus	95.0± 4.4	93.5± 1.2
Overall		96.7± 1.3	95.8± 0.9

Note: The first column shows specific anatomical structures of mouse hippocampus. The second and third columns show the percentages of CCK or GABA immunopositive CCK&Dlx5/6-GFP-expressing neurons relative to total CCK&Dlx5/6-GFP-expressing neurons in corresponding structures, respectively. The number is presented in percentages as mean ± SD. Total cell counts for CCK or GABA staining are 2489 and 2723 neurons from 3 mice, respectively. Abbreviations for regions and layers are as follows: cornu Ammonis 1 (CA1), cornu Ammonis 3 (CA3), dentate gyrus (DG), stratum oriens (S.O.), stratum pyramidale (S.P.), stratum radiatum (S.R.), stratum lacunosum moleculare (S.L.M), stratum lucidum (S.L.U), molecular layer (ML), and granule cell layer (GCL).

Supplemental Table 2. Average densities of neurochemical marker-positive neurons in different mouse hippocampal laminae

Cells /mm^2	CA1				CA3					DG		
	S.O.	S.P.	S.R.	S.L.M.	S.O.	S.P.	S.L.U.	S.R.	S.L.M.	MCL	GCL	Hilus
(CCK&Dlx5/6)+	68.9± 7.4	207.5± 41.2	53.2± 10.2	131.0± 34.8	41.9± 8.5	128.2± 28.5	67.9± 10.9	86.5± 17.4	111.5± 47.6	17.6± 4.7	119.9± 16.1	49.9± 2.5
PV+	51.2± 14.5	134.6± 23.5	10.8± 2.6	4.6± 2.7	39.4± 4.0	113.9± 36.1	29.7± 14.8	23.5± 3.7	0	1.5± 0.6	54.4± 8.9	24.8± 13.5
CR+	22.5± 12.4	92.1± 18.5	24.6± 5.9	127.4± 29.9	9.1± 8.9	35.5± 14.8	32.4± 8.5	23.6± 17.5	64.6± 36.3	58.0± 19.5	322.4± 73.9	337.9± 143.3
SOM+	112.2± 10.4	39.3± 15.1	12.9± 2.1	2.9± 1.1	56.1± 14.3	32.1± 4.0	93.1± 4.4	35.7± 4.8	2.1± 3.7	0.8± 0.8	0	158.3± 22.6
VIP+	14.5± 5.3	50.9± 2.1	10.2± 2.3	17.8± 1.5	8.3± 2.7	32.1± 7.4	33.2± 10.4	13.0± 2.4	2.7± 4.6	3.2± 3.9	15.6± 3.7	0

Note: the table shows average densities of specific types (neurochemical maker-positive) hippocampal neurons, including PV+, CR+, SOM+, and VIP+, in specific anatomical structures of mouse hippocampus. The numbers are presented as mean ± SD cells/mm^2 (Cell Density). (CCK&Dlx5/6)+ cell density calculated by using CCK&Dlx5/6-GFP transgenic mice (total cell counts for (CCK&Dlx5/6)+: 9718 from 3 mice). PV+, CR+, SOM+, and VIP+ cell densities calculated by immunostaining (total cell counts for PV+, CR+, SOM+, and VIP+ are 1805, 1700, 884, 383 from 3 mice, respectively).

Supplemental Table 3. Average cell densities of inhibitory neurons that are (CCK&Dlx5/6)+ with other neurochemical markers in different hippocampal laminae

Cells /mm^2	CA1				CA3					DG		
	S.O.	S.P.	S.R.	S.L.M.	S.O.	S.P.	S.L.U.	S.R.	S.L.M.	MCL	GCL	Hilus
(CCK&Dlx5/6)+ and PV+	14.6± 6.6	63.3± 7.2	2.2± 1.7	0	2.7± 0.8	22.9± 4.2	2.4± 2.2	1.3± 0.3	0	1.0± 0.5	22.7± 2.8	0
(CCK&Dlx5/6)+ and CR+	2.0± 1.5	10.9± 6.1	0.6± 0.7	3.4± 1.1	0	1.5± 2.6	0	2.0± 0.2	0	0.5± 0.5	3.8± 3.9	0
(CCK&Dlx5/6)+ and SOM+	10.7± 2.9	3.5± 3.2	1.7± 0.6	0	1.2± 1.1	0	28.8± 10.6	2.8± 2.9	0	0	0	6.3± 6.3
(CCK&Dlx5/6)+ and VIP+	3.8± 3.4	18.3± 3.0	2.7± 2.5	1.8± 1.6	1.4± 1.8	5.1± 3.5	9.8± 4.9	1.1± 1.2	0	0	2.7± 2.4	0

Note: the table shows average densities of neurons that co-express (CCK&Dlx5/6)+ and PV+, (CCK&Dlx5/6)+ and CR+, (CCK&Dlx5/6)+ and SOM+, and (CCK&Dlx5/6)+ and VIP+ in specific anatomical structures of mouse hippocampus, respectively. The numbers are presented as mean ± SD cells/mm^2 (Cell Density). Cell densities are calculated by using CCK&Dlx5/6-GFP transgenic mice coupled with immunostaining on PV+, CR+, SOM+, and VIP+, respectively (total cell counts for (CCK&Dlx5/6)+ and PV+, (CCK&Dlx5/6)+ and CR+, (CCK&Dlx5/6)+ and SOM+, and (CCK&Dlx5/6)+ and VIP+ are 516, 54, 71, and 83 from 3 mice, respectively).

Supplemental Table 4. Percentages of inhibitory neurons with neurochemical co-expression in different hippocampal regions.

%	CA1				CA3					DG		
	S.O.	S.P.	S.R.	S.L.M.	S.O.	S.P.	S.L.U.	S.R.	S.L.M.	MCL	GCL	Hilus
((CCK&Dlx5/6)+ and PV+) / (CCK&Dlx5/6)+	20.3± 7.6	31.4± 6.1	3.5± 3.3	0	7.0± 0.5	20.6± 2.9	4.1± 3.6	1.8± 0.8	0	6.4± 2.5	19.3± 6.0	0
((CCK&Dlx5/6)+ and PV+) / PV+	28.8± 8.2	48.1± 7.8	18.2± 15.3	0	7.6± 0.8	21.4± 6.8	10.8± 10.1	5.9± 2.1	0	70.0± 26.5	41.7± 2.1	0
((CCK&Dlx5/6)+ and CR+) / (CCK&Dlx5/6)+	3.4± 3.0	4.8± 2.8	1.1± 1.2	3.5± 2.0	0	2.1± 3.6	0	2.1± 0.5	0	2.3± 2.1	2.7± 2.6	0
((CCK&Dlx5/6)+ and CR+) / CR+	8.0± 3.4	12.5± 8.8	3.1± 3.8	3.7± 1.8	0	3.7± 6.4	0	12.3± 7.7	0	1.2± 1.1	1.0± 1.0	0
((CCK&Dlx5/6)+ and SOM+) / (CCK&Dlx5/6)+	14.28± 2.2	1.65± 1.4	3.49± 1.7	0	2.32± 2.0	0	31.92± 10.9	2.66± 3.1	0	0	0	12.5± 12.5
((CCK&Dlx5/6)+ and SOM+) / SOM+	9.79± 2.9	8.11± 5.4	13.57± 1.7	0	1.48± 1.3	0	29.38± 11.0	7.84± 9.0	0	0	0	4.37± 4.8
((CCK&Dlx5/6)+ and VIP+) / (CCK&Dlx5/6)+	6.4± 5.4	8.7± 0.3	4.4± 2.8	2.0± 1.8	3.2± 3.5	4.4± 3.5	19.7± 17.8	1.8± 1.6	0	0	2.2± 2.0	0
((CCK&Dlx5/6)+ and VIP+) / VIP+	26.8± 14.1	35.5± 6.0	21.6± 10.4	10.6± 9.2	26.4± 35.4	15.7± 9.1	31.1± 17.1	11.4± 10.3	0	0	17.9± 15.6	0

Note: The table shows percentages of the number of neurochemical co-expressing neurons (e.g. (CCK&Dlx5/6)+ and PV+) relative to the number of neurons labeled by single marker in each corresponding anatomical structures in mouse hippocampus. The data presented as mean ± SD in percentage.

Supplemental Table 5. Average cell densities of inhibitory neurons that genetically are PV+ and (CCK&Dlx5/6)+ in CA1 of hippocampal laminae

Cells /mm²	CA1			
	S.O.	S.P.	S.R.	S.L.M.
(CCK&Dlx5/6)+	45.5± 10.8	153.0± 39.1	20.5± 10.1	26.9± 15.4
PV+	39.3± 15.3	195.1± 47.0	4.4± 4.3	3.1± 8.2
PV+ and (CCK&Dlx5/6)+	9.2± 6.8	79.9± 25.0	0.6± 0.9	0.7± 1.5

	Hippocampus	Cortex
((CCK&Dlx5/6)+ and PV+) / (CCK&Dlx5/6)+	10.9± 0.7%	30.9± 7.0%
((CCK&Dlx5/6)+ and PV+) /PV+	7.3± 1.7%	2.2± 0.2%

Note: the table shows average densities of neurons that are PV+ and (CCK&Dlx5/6)+ in CA1 of mouse hippocampus. The numbers are presented as mean ± SD cells/mm^2. Cell densities calculated using PV-tdTomato; CCK&Dlx5/6-GFP transgenic mice where PV cells express tdTomato and (CCK&Dlx5/6)+ inhibitory cells express GFP. (The total cell count for PV+ and (CCK&Dlx5/6)+ is 1278 cells from 3 mice). The table also shows percentages of co-expressed PV+ and (CCK&Dlx5/6)+ neurons relative to the number of neurons labeled by single marker PV or (CCK&Dlx5/6)+. Cell counts were generated by surveying labelled neurons in hippocampus and surrounding cortical areas in PV-tdTomato; CCK&Dlx5/6-GFP transgenic mice with fluorescently tagged PV+ and (CCK&Dlx5/6)+ cells. The data presented in percentage as mean ± SD and represented a total of 6968 PV+ cells and 933 (CCK&Dlx5/6)+ cells. Abbreviations for regions and layers are as follows: cornu Ammonis 1 (CA1), stratum oriens (S.O.), stratum pyramidale (S.P.), stratum radiatum (S.R.), and stratum lacunosum moleculare (S.L.M).

Supplemental Table 6. Primary antibodies used for immunohistochemistry

Antigen	Immunogen	Manufacturing Details	Working dilution
CCK	Synthetic sulfated CCK-8 conjugated to KLH	Sigma-Aldrich (St. Louis, MO), rabbit polyclonal , C2581	1:500
CR	Recombinant human calretinin	Swant (Bellinzona, Switzerland), rabbit polyclonal, 7699/3H	1:500
GABA	(α-Aminobutyric Acid)-BSA	Sigma-Aldrich (St. Louis, MO), rabbit polyclonal, A2052	1:1000
GFP	Recombinant GFP protein	Aves Labs, Inc. (Tigard, OR), Chicken polyclonal, GFP-1020	1:500
PV	Rat muscle parvalbumin	Swant (Bellinzona, Switzerland), rabbit polyclonal, PV-25	1:1000
PV	Rat muscle parvalbumin	Swant (Bellinzona, Switzerland), goat polyclonal, PG-214	1:1000
SOM	Synthetic 1–14 cyclic somatostatin	Millipore/Chemicon (Temecula, CA), rat monoclonal, MAB354	1:200
VIP	Porcine VIP conjugated to bovine thyroglobulin with carbodiimide	ImmunoStar, Inc. (Hudson, WI), rabbit polyclonal, 20077	1:500
COX6a2	C-terminus region of human COX6A2	Novus (Centennial CO) Rabbit polyclonal, 31112	1:500
