# Supplementary material for: Anatomical and molecular characterization of parvalbumin-cholecystokinin co-expressing inhibitory interneurons: implications for neuropsychiatric conditions
Source: Mol Psychiatry. 2023 Jul 13;28(12):5293–308. doi: 10.1038/s41380-023-02153-5 (PMC11041731; doi:10.1038/s41380-023-02153-5)
Supplement: Supplementary file 6 — Supplemental Material and Methods [file 41380_2023_2153_MOESM6_ESM.pdf]

Supplemental Materials and Methods

Animals

All animals were handled and experiments were conducted according to the National Institutes of Health guidelines for animal care and use, and procedures were approved by the Institutional Animal Care and Use Committee of the University of California, Irvine. Mice of 8-12 weeks old (either sex) were used for experiments (4-6 weeks old for electrophysiology), and had free access to food and water in their home-cages before sacrifice. The animal numbers and cell samples were calculated based on power analysis or estimation from previous studies, including our own published studies. The "a priori" criteria are defined before executing experiments for rational inclusion/exclusion of data, as established in our published studies. Specifically, if a technique failed (injection target was missed, for example) that data was excluded. Whenever possible, mice were randomly assigned to experimental vs. control groups with matched age and sex. Experiments were not blinded during data acquisition, but imaging and behavioral data analysis were performed blind to treatment.

The CCK&Dlx5/6-GFP (CCK-Cre:Dlx5/6-Flp:GFP) triple transgenic mouse was generated by first crossing the CCK-ires-Cre mouse line (Jackson Laboratories, stock no. 012706, generated by Z.J. Huang, CSHL) [1] with the Dlx5/6-Flp mouse line (Jackson Laboratories, stock no. 010815, generated by G. Fishell, Harvard) [2]. A second cross used the RCE-dual mouse line (R26R CAG-boosted EGFP; Jackson Laboratories, stock no. 032036, generated by G. Fishell, Harvard) [3], a double STOP EGFP reporter, bred with the Cre+/Flp+ progeny (termed as CCK-Cre:Dlx5/6-Flp) generated from the first cross. In the CCK&Dlx5/6-GFP mouse, all CCK&Dlx5/6+ interneurons express EGFP as they

undergo both Cre and Flp recombination.

The PV-Cre: tdTomato double transgenic mice were obtained by crossing the PV-Cre mice (Jackson Laboratories, stock no. 008069, generated by S. Arbor, FMI) [4] to an Ai9 reporter line (Rosa-CAG-LSL-tdTomato; Jackson Laboratories, stock no. 007905, generated by H. Zeng, Allen Brain Institute) [5], so that parvalbumin positive neurons express the tdTomato fluorescent protein. By crossing the PV-Cre: tdTomato double transgenic mice with the RCE-dual mouse line, we generated a RCE-dual; PV-Cre:tdTomato mouse line mice that could be crossed with CCK-Cre:Dlx5/6-Flp mice to generate the final offspring (PV-tdTomato; CCK&Dlx5/6-GFP).

Mice used for behavioral experiments were hybrid offspring of homozygous CCK-IRES-Cre (Jackson Laboratories, stock no. 012706) and Pvalb-2A-Flp (Jackson Laboratories, stock no. 021191, generated by H. Zheng, AI) [5] parents, resulting in heterozygous mice expressing CCK-targeted expression of Cre recombinase and Pvalb-targeted expression of Flp recombinase proteins.

Mice used for sn-RNA-Seq experiments were a results of crossing PV-Cre mice (Jackson Laboratories, stock no. 008069, generated by S. Arbor, FMI) [4] with R26-CAG-LSL-Sun1-sfGFP-Myc mice (Jackson Laboratories, stock no. 030952, generated by J. Nathans, JHU) [6]. We term the resulting progeny PV-Cre; cSUN1, and used the GFP-tagged nuclei of PV interneurons for downstream sn-RNA-Seq applications to measure *CCK* mRNA expression.

Rats are the Sprage Dawley strain and were purchased from Charles River (strain #400). Both females and male rats were used and were 3-6 months old. Macaque monkey hippocampal sample were collected from Dr. Greg Horwitz's laboratory at the University

of Washington per their protocol approval, and animals were originally obtained from the Washington National Primate Research Center.

Tissue preparation

For immunochemical staining experiments, the mice or rats were deeply anesthetized with Uthasol (sodium pentobarbital, 100 mg/kg, i.p.) and transcardially perfused with 5 ml 0.1M phosphate buffered saline (PBS, pH 7.3–7.4), followed by 25 ml (50 ml for rats) 0.1M PBS containing 4% paraformaldehyde. The brains were removed and left in 4% paraformaldehyde overnight, then transferred into 30% sucrose in 0.1M PBS in the next day. The brain was sectioned coronally in 25μm thickness on a freezing microtome (Leica SM2010R, Germany). Selected mouse, rat and monkey hippocampal sections were used for immunohistochmistry experiments and quantitative analysis.

Immunohistochemistry

To stain tissue sections with antibodies, conventional fluorescent immunohistochemistry was performed. Free-floating sections were rinsed 5-6 times with PBS with 0.1% Triton X, and incubated in a blocker solution for 2 hours at room temperature. The blocker solution contains 10% normal donkey serum, 2% bovine serum albumin, and 0.25% Triton X in PBS. Sections were then incubated with the primary antibody in blocker solution at the appropriate dilution for 36 hours at 4 ^{o}C. After the primary antibody incubation, sections were rinsed thoroughly with PBS (or working buffer: 10% blocker and 90% PBS; 6 times), and then incubated with an appropriate secondary antibody in the blocker solutions for 2 hours at room temperature. After the secondary

antibody solution was rinsed off, sections were counterstained with 10 μM 4'-6-diamidino-2- phenylindole (DAPI; Sigma-Aldrich) for 10 minutes to help distinguish hippocampal subfields and laminar structures. Finally, sections were rinsed and wet-mounted, and were directly coverslipped with the mounting medium Vectashield (H- 1000, Vector, Burlingame, CA) after dried at room temperature.

All primary antibodies used in our staining experiments are commercially available from major companies (Supplemental Table 6). For characterizing different type of inhibitory neurons, we stained mouse hippocampal sections against calcium binding protein parvalbumin (PV) and the neuropeptide cholecystokinin (CCK). In order to determine GABAergic neurons, we stained brain slices against γ-Aminobutyric acid (GABA). We used GFP antibody to amplify the EGFP signals in CCK-dual mice.

Based on technical information from Swant, the specificity of goat anti-PV antibody is determined by immunoblots of brain homogenate; both rabbit anti-PV and goat anti-PV antibodies stain a subpopulation of neurons in the normal brain with high efficiency, but do not stain the brain tissue of PV knockout mice. Immunoabsorption tests of the PV antibodies have been carried out by Xu et al., 2010 to confirm specificity [7]. According to the product information provided by Sigma-Aldrich, rabbit anti-Cholecystokinin (26-33) (CCK-8) specifically stains CCK- and gastrin-containing cells in formalin-fixed, paraffin-embedded sections of human stomach (neuroendocrine cells). The antibody recognizes sulfated CCK-8 in radioimmunoassay, and inhibited with sulfated CCK-8 and with human gastrin I in specific staining. Cross-reactivity is observed with unsulfated CCK-8 and caerulein. Low cross-reactivity is observed with human gastrin I, CCK (30-33) and human Big Gastrin. According to technical information from Millipore/ Chemicon, the rabbit anti-

GAD65/67 antibody is purified using Ammonium sulfate precipitation, and the specificity is characterized by both Western blot and immunohistochemistry using the tissue from human, mouse, rat, and feline. In Western blot of rat/mouse whole brain lysate, the antibody is able to efficiently label both GAD65 and GAD67 isoforms in a clear manner. The GFP antibody is analyzed by western blot analysis (1:5000 dilution) and immunohistochemistry (1:500 dilution) using transgenic mice expressing the GFP gene (Aves Labs data sheet).

The secondary antibodies, Cy3-conjugated or Alexa Fluor 488–conjugated donkey anti-rabbit IgG (Jackson ImmunoResearch, West Grove, PA; 711-165-152 or 711-225-152,1:200 dilution) were used for fluorescent visualization of the immunostaining. None of the observed labeling was due to nonspecific binding of secondary antibodies or autofluorescence in the fixed tissue because sections labeled with secondary antibodies alone showed no detectable labeling.

Image data acquisition and analysis

After immunostaining, the sections were examined, and low- and high-power images were acquired by using an Automated Slide Scanning and Analysis software (Metamorph, Inc.) in a high-capacity computer coupled with a fluorescent BX61 Olympus microscope and a high-sensitive CCD camera (Hamamatsu Photonics, Tokyo, Japan). Under a 10X objective, we were able to obtain sufficient-resolution images suitable for all subsequent computer-based analyses. We also imaged labeled cells in selected sections with a confocal microscope (LSM 700, Carl Zeiss Microscopy, Nussloch, Germany). Image stitching, overlaying, cell counting and further imaging analysis were completed by

using Metamorph/Adobe Photoshop imaging and analysis tools. Quantitative examinations across the series of sections were conducted for complete and unbiased analyses of immunohistochemistry over different cell types.

Volumetric Confocal and Light Sheet Imaging

Preparation of clearing and immunostaining pretreatment solutions (PEGASOS, TESOS and iDISCO).

Decolorization solutions: Quadrol (N,N,N',N'- Tetrakis (2-hydroxypropyl) ethylenediaminel) (Sigma-Aldrich 122262) was diluted with distilled H_2O to a final concentration of 25% v/v. Gradient tB delipidation solution: Pure tert-Butanol (tB) (Sigma-Aldrich 360538) was diluted with distilled H_2O to prepare gradient delipidation solutions at 30% v/v, 50% v/v and 70% v/v. Quadrol was then added with 3~5% w/v final concentration to adjust the pH to above 9.5. Staining pretreatment solutions: PBS/0.2% Triton X-100(PTX.2) was composed of 0.2% Triton X-100 in 0.01M PBS. Permeabilization solution was composed of 0.2% Triton X-100, 20% DMSO, 0.3M glycine in 0.01M PBS. Blocking solution was composed of 0.2% Triton X-100, 10% DMSO, 3% donkey serum in 0.01M PBS. Staining solutions: PBS/0.2% Tween-20 with 10 μg/ml heparin (PTwH) was composed of 0.2% Tween-20 with 10 μg/ml heparin in PBS. Primary antibody was diluted in PTwH with 5% DMSO, 1% Donkey Serum depending on the recommended dilution ratio. Secondary antibody was diluted in PTwH with 1% Donkey Serum depending on the recommended dilution ratio. Centrifuging antibody solution at 20,000g for 10 minutes can prevent formation of precipitates in the sample. Alternatively, the solution was syringe-filtered at 0.2μm. tB-PEG dehydration solution: Dehydrating solution was composed of

70% v/v tert-Butanol, 27% v/v PEG methacrylate Mn 500 (PEGMMA500) and 3~5% w/v Quadrol. BB-PEG clearing medium (Refractive index R.I. 1.543) (For LightSheet microscope imaging): BB-PEG was prepared by mixing 75% v/v benzyl benzoate (BB, Sigma-Aldrich W213802) and 25% v/v PEGMMA500 supplemented with 3~5% w/v Quadrol together. The fresh medium was a colorless liquid with low viscosity and turned slightly yellow in a week. BB-BED Clearing medium (Refractive index R.I.1.552) (For confocal microscope imaging): BB-BED was composed of 47% (v/v) benzyl benzoate (BB, Sigma-Aldrich W213802), 48% (v/v) of Bisphenol-A ethoxylate diacrylate Mn 512 (BED512) (Sigma-Aldrich 412090), 5% (v/v) of Quadrol and add additional 2% w/v of 2-Hydroxy-4'-(2-hydroxyethoxy)-2-methylpropiophenone (Sigma Aldrich 410896) as the UV initiator.

Clearing and immunostaining procedures (PEGASOS, TESOS and iDISCO).

The clearing and staining procedures are published [8-10]. The whole process was performed on 37°C shaker. Overnight or 24 hours postfixed brain tissues were washed in PBS for 1hr, twice. Samples were then immersed into decolorization solution for 2 days with daily changing. Following that, samples were placed in gradient tB delipidization solution for 2 days. 30% tB for ~4 hrs, 50% tB for ~6 hrs and 70% tB for the remaining time. Samples were then washed in PTX.2 for 1 hr, twice, followed by permeabilization solution for 2 days. After that, the pretreated samples were immersed in blocking solution for another 2 days, then washed in PTwH 1 hr twice and incubated in staining solution based on recommended dilution ratio for 5 days or longer (based on sample size). Samples were then wash in PTwH 5 times per day for 2 days and switched to secondary

antibody solutions. Samples were finally washed in PTwH 5 times per day for 2 days. A second round of gradient tB delipidation on the samples was performed using 30%, 50% and 70% v/v tB for 2 days. Later, samples were incubated in tB-PEG dehydration solution for 1 to 2 days with daily changing. Samples were then switched to new containers with clearing medium BB-PEG for 2 days. Samples were then imaged under the LightSheet microscope. After LightSheet microscope imaging, the samples were immersed in BB-BED for another 2 days. Samples and the BB-BED were then cured and turned into organogels using UV light and were then ready to be sectioned and imaged using confocal microscope.

LightSheet microscopy and Confocal imaging and animation

2 mm PEGASOS cleared brain slices fluorescent images were acquired with the Cleared Tissue LightSheet (CTLS) microscope (3I Inc) (visible laser lines: 488,561,670 nm). The samples were immersed in BB-PEG clearing medium and scanned. A tiling light-sheet tiled at multiple positions within the field of view was used to illuminate the sample, and the sample was scanned with a 1.5×/0.25NA objective axially at a ~1 μm step size to image the sample in 3-D. All raw image data were collected in a lossless 16-bit TIFF files. 3D reconstruction images were generated using Slidebook (3I) and Imaris (Bitplane).

The 2 mm TESOS cleared brain slices fluorescent images were acquired with the confocal laser scanning microscope, FV3000 (Olympus) (visible laser lines: 405, 488,561,670 nm). Samples were embedded in the BB-BED and cured using UV light. The liquid BB-BED and the sample turned into hard organogel and was mounted on a two-part magnetic kinematic base2. The organogel plus kinematic base was then mounted on

the rotary microtome and sectioned (5 µm or 10 µm / slice) until the sample top face was exposed. After sectioning, an extra drop of liquid BB-BED clearing medium was added on the sample and a coverslip was placed on it. The samples were imaged with confocal microscope and 60x oil objective lens (Olympus UPLXAPO 60X Oil Immersion Objective, Stock #14-909) as regular slides. The imaging depth of each stack is ~120um with ~0.4um z step. All raw image data were collected in a OIR file and converted into .ims format (Imaris Converter Bitplane). 3D rendering and animation were accomplished using Imaris (Bitplane).

Single-cell RT-PCR

Coronal brain slices containing the hippocampus of CCK-Cre;Dlx5/6-Flp;GFP (CCK&Dlx5/6-GFP) mice were used to identify GFP-expressing interneurons. Fluorescent cells were identified and selected via the fluorescent mode of an Olympus microscope (BX51WI); subsequently, "break-in" was performed under visual control aided by infrared differential interference contrast (IR‐DIC) video monitoring through the microscope and a Qimaging video camera (RETIGA-2000). Glass electrodes, filled with an internal solution and 0.1% biocytin, were used to visualize the cells before and after extraction of cellular contents. To extract the contents a negative pressure was applied and the extraction was as complete as possible. The cellular contents of individually identified neurons were then placed into individual PCR tubes, by breaking off the tip of the electrode containing the contents, pre-prepared for reverse transcription (RT).

Cellular mRNA (9uL of cellular contents) was converted to cDNA using Superscript® III reverse transcriptase (Thermo Fisher Scientific, Waltham, MA) according

to the manufacturer's instructions. Briefly, an annealing step was first conducted at 65ºC for 5 minutes with Oligo(dT) primers and a dNTP mix. Then RT was performed at 42ºC for 6 hours, 70ºC for 15mins, and then 4ºC using an RT buffer, $MgCl_2$, DTT, RNAseOUT, and the reverse transcriptase. cDNA was then amplified using two successive PCR reactions. The first PCR was conducted with standard denaturing (95ºC), annealing (50ºC) and extension (72ºC) steps using 5uL of the RT product, DNA polymerase, PCR buffer, dNTPs, $MgSO_4$, water and a final concentration of .3µM of each primer. The second PCR was conducted with standard denaturing (95ºC), annealing (50ºC) and extension (72ºC) steps using 1uL of the first PCR product, DNA polymerase, PCR buffer, dNTPs, $MgSO_4$, water and a final concentration of .3µM of each primer. To ensure accuracy of the PCR product, different primer sets were used for the same gene for each PCR. For CCK mRNA (NM_031161.3) for the 1st PCR primers were: Sense, 5'-TGTCTGTGCGTGGTGATGGC -3' and Antisense, 5'-GCATAGCAACATTAGGTCTGGGAG -3'. For the 2nd PCR primers were: Sense, 5'-ATACATCCAGCAGGTCCGCAA -3' and Anti, 5'- CAGACATTAGAGGCGAGGGGT -3'. For PV mRNA (NM_013645.3) for the 1st PCR primers were: Sense,5'-GCCTGAAGAAAAAGAACCCG-3' and Antisense, 5'-AATCTTGCCGTCCCCATCCT -3'. For the 2st PCR primers were: Sense, 5'- CGGATGAGGTGAAGAAGGTGT-3' and Antisense, 5'- TCCCCATCCTTGTCTCCAGC-3'. PCR products were then separated on agarose gels using standard electrophoresis along with a DNA ladder to identify bands. Gels were subsequently stained with ethidium bromide for visualization and imaging for analysis of the molecular identity of each cell.

sn-RNA-Seq of Hippocampus (PV-Cre; cSUN1-EGFP mice)

We first performed a nuclei preparation from PV-Cre; cSUN1 [6] mice (PV-Cre; R26-CAG-LSL-Sun1-sfGFP-Myc). Hippocampi from both hemispheres was harvested on ice and then dounced in 1 mL of NIM-DP-L buffer (contains sucrose, KCl, MgCl2, Tris-HCl, DTT, protease inhibitor, RNAseOUT, and Triton-X 100). We use the loose plunger first (usually 5-10 strokes), then the tight plunger (usually 15-25 strokes). We then filter the homogenate with a 30 μm filter. Nuclei are then centrifuged (200 rcf, 10 min, 4˚C) and the supernatant is aspirated. The pellet is resuspended and centrifuged again. Then the pellet is resuspended in sorting buffer (contains 1XPBS, EDTA, BSA and RNAseOUT) and is filtered again. We then used a Sony (SH800S model) sorter, and sorted EGFP+ nuclei directly into RT buffer.

The Chromium Next GEM Single Cell 3' GEM, Library & Gel Bead Kit v3.1 was used for scRNA-seq library construction. In this protocol, thousands of cells are partitioned into nanoliter size Gel Bead-In-Emulsions using the 10x Chromium controller. The emulsions are transferred to PCR strip tubes and first strand cDNA synthesis using reverse transcriptase occurs in a thermal cycler. The cDNA that is generated from a single cell all share the same barcode and each transcript is labeled with unique molecular identifier. After the first strand cDNA synthesis the reaction is cleaned up using silane magnetic beads. The full length barcoded cDNA is then amplified by PCR to generate enough material for Illumina sequencing library construction. The cDNA is then enzymatically fragmented and size selected. Following end repair, A-tailing and adapter ligation a final PCR enriches the library fragments with Illumina adapters. The sequencing

was completed on a Illumina NovaSeq 6000 with 28 bases of read 1, 8 bases of index read and 100 bases in read 2.

For analysis the dataset consisted of one set of snRNA-seq expression profiles sequenced from curated Pvalb expressing interneurons, consisting of 2,387 cells. Analysis of snRNA-seq data was performed using the Seurat v3 R package. Quality control consisted of the elimination of cells exhibiting fewer than 200 or more than 2,500 features (non-zero entries in the gene expression matrix), which eliminated 80 cells. An additional measure involved removing data corresponding to genes not expressed in any cells. Next, the gene expression matrix was log-normalized, and the top variable features were identified, and the expression matrix was scaled to have mean 0 variance 1 expression across cells. The top 2000 variable features were used in dimensionality reduction which consisted first of PCA (principle component analysis), followed by a UMAP (Uniform Manifold Approximation and Projection) projection for visualization. The elbow plot method was used to identify the “dimensionality” of the data, and 8 dimensions were used for the PCA projection, cell clustering, and input to the UMAP algorithm.

Electrophysiology and Cell Identification

4-6 week-old animals were deeply anesthetized with isoflurane. After decapitation brains were quickly removed to make acute brain slices. A vibratome (VT1200S, Leica Systems) was used to cut 300 μm-thick coronal brain slices from hippocampus. Slices were cut in ice-cold sucrose containing artificial cerebrospinal fluid (ACSF) (in mM: 85 NaCl, 75 sucrose, 2.5 KCl, 25 glucose, 1.25 NaH_2PO_4, 4 $MgCl_2$, 0.5 $CaCl_2$, and 24 $NaHCO_3$). Slices were incubated for at least 30 min with normal ACSF (in mM: 126 NaCl,

2.5 KCl, 26 $NaHCO_3$, 2 $CaCl_2$, 2 $MgCl_2$, 1.25 NaH_2PO_4, and 10 glucose) in an interface holding chamber aerated with 95% O_2 / 5% CO_2 at 32°C for at least 30 minutes before they were transferred to a recording chamber containing ACSF bubbled with 95% O_2 / 5% CO_2.

Oxygenated ACSF at room temperature was perfused into the slice recording chamber through a custom-designed flow system driven by pressurized 95% O_2/5% CO_2 (3 psi) at roughly 2 ml/min. GFP- and/or td-tomato- expressing interneurons were first identified and selected via the fluorescent mode of an Olympus microscope (BX51WI); subsequently, whole-cell patch-clamp recordings were performed under visual control aided by infrared differential interference contrast (IR-DIC) video monitoring through the microscope and a Qimaging video camera (RETIGA-2000). Glass recording electrodes (4–6 MΩ resistance) were filled with an internal solution consisting of 126 potassium-gluconate, 4 KCl, 10 HEPES, 4 ATP-Mg, 0.3 GTP-Na, and 10 phosphocreatine (pH 7.2-7.3, 290-300 mosM). 0.1% biocytin was added for post hoc morphological analysis. Once stable whole-cell recordings were achieved, basic electrophysiological properties were examined through hyperpolarizing and depolarizing current step injections. Electrophysiological data were acquired with a Multiclamp 700B amplifier (Molecular Devices), data acquisition boards (models PCI MIO 16E-4 and 6713, National Instruments), and custom modified version of Ephus software [11]. Data were digitized at 10 kHz. No correction was made for the liquid junction potential.

Hyperpolarizing and depolarizing current pulses (duration: 1000 ms) were injected to examine each cell's basic electrophysiological properties. Firing rate, spike frequency adaptation, as well as shapes of single spikes elicited by depolarizing current pulses at

threshold strength. Spike frequency adaptation index was calculated as the ratio of the first interspike interval and the last interspike interval of a spike train evoked by a depolarizing current step (~300 pA) of 1000 ms duration. Briefly, the action potential threshold was determined as the intersection between linear fits to the gradual depolarizing phase preceding the action potential (from 10 ms preceding the action potential peak) and the rapidly rising phase of the action potential (to the peak of the derivative of membrane potential). Action potential width was defined as the spike width at its half height. Afterhyperpolarization (AHP) amplitude was defined as the difference between action potential threshold and the most negative membrane potential attained during the AHP. AHP time is defined as the time from the action potential peak to the trough of the AHP. We only included cells with resting potentials more negative than −50 mV and access resistance less than 40 MΩ for electrophysiological analyses; spike shape analyses were restricted to cells that had action potential amplitudes of at least 40 mV.

After recordings, the brain slices were fixed in 4% PFA overnight, then transferred to 30% sucrose solution in PBS. The slices were stained for biocytin with 1:1,000 Cy3-conjugated streptavidin (016-160-084, Jackson ImmunoResearch) to reveal the morphology of the recorded cell. GFP and td-tomato fluorescence were enhanced with GFP and td-tomato antibodies (chicken x EGFP 1:500, rabbit x dsRed 1:300, secondary antibodies (donkey x chicken AF488 green1:200, donkey x rabbit cy3 red 1:200). Cell morphology was examined by confocal microscopy, which also confirmed that each recorded cell was indeed the GFP- and/or td-tomato- expressing interneuron. Imaris commercial software was used to characterize the dendrite length, area and volume of PV+/CCK&Dlx5/6+, PV-/CCK&Dlx5/6+, and PV+/CCK&Dlx5/6- interneurons.

Analysis of Multiome Data

After production, sn-RNA-Seq samples underwent QC individually. Cells were required to exhibit >300 individual transcripts, and to have a mitochondrial percentage less than 1%. Doublets were identified using scDblFinder (1.10.0), and ~10% of cells were removed as doublets. Samples were normalized using scTransform and integrated using Harmony (0.1.1) after PCA dimensionality reduction. Clusters were identified using the Louvain algorithm and reduced to two dimensions via UMAP. Interneurons were identified by expression of GAD1/2. The combined sample was subset to the set of interneurons. Primary analysis was performed using Seurat (4.2.1).

Next, dimensionality reduction and integration were recomputed, and new clusters were identified. These were subset into the major interneuron types (Meis2, PV, SST, Lamp5, Vip, Sncg). Vipr2 expressing PV+ interneurons, a transcriptional profile commonly identified with chandelier cells, were retained as PV+ interneurons. PV+ interneurons were identified as clusters strongly expressing PVALB, and retained for further analysis. A total of n = 737 hippocampal PV+ interneurons were identified.

At this point, the ATAC samples were subset using the cell IDs of retained PV+ interneurons. Using Signac (1.9.0), common peak sets were identified, and the samples were merged. Transcription start site enrichment was computed, and a threshold of 3.5 (in our experience, Signac produces lower than average TSS enrichment scores) was utilized for QC, leaving a total of n = 632 cells for combined analysis. For CCK peak to gene analysis, peaks were recalculated using MACS2 (2.2.7.1). For integrated analysis, CCK+ cells were designated as those expressing at least one count of CCK.

Differential expression analysis was performed using both Seurat and DESeq2. In the latter case, the adjustments suggested for single-cell analysis were applied. Dropout reads were imputed using MAGIC (2.0.3), gene module analysis was performed using WGCNA (1.72-1), and gene ontology/KEGG pathway ontology was performed using a combination of topGO (2.48.0) and gProfiler2 (0.2.1) using the org.Mm.eg.db package (3.15.0) and the EnsDb.Mmusculus.v79 (2.99.0) package. Gene linkages were computed in Signac (function FindLinks) using suggested parameters, except the distance from the TSS was allowed to increase by a factor of 100.

Analysis of Mined Transcriptomic Data

Raw sn/sc-RNA-Seq was analyzed from publicly available datasets. A total of five datasets were analyzed, consisting of two scRNA-seq (10x v3 and SMART-Seq) sample sets covering the isocortex and hippocampal formation, and three snRNA-seq (1 10x v2, 2 10x v3) samples taken from the primary motor cortex of the adult mouse [12]. Individual datasets were analyzed via the same pipeline, on the Seurat platform, consisting of log-normalization, filtering of cells with outlying numbers of features, identification of variable genes, scaling, and PCA dimensionality reduction [13]. Interneurons were identified based on GAD1 and GAD2 expression, as well as provided taxonomy from stated publications. This resulted in 21,991 cells from the Allen SMART-seq dataset (labeled 2019Allen), 96,323 cells from the Allen 10x dataset (labeled 2020Allen), 10,944 cells from the BICCN 10x v2 dataset (labeled 2021AIBS_v2), 6,817 from the first BICCN 10x v3 dataset (labeled 2021AIBS_v3), and 18,093 cells from the second BICCN 10x v3 dataset (labeled 2021BROAD_v3). Of these, a total of 28,951 were labeled as PV interneurons, and 7,053

were labeled as SNCG. Datasets were integrated via Harmony[14]. Results corroborated previous experiments showing interneuron cell types were shared across brain regions, motivating combined analysis across regions[12]. Dropout gene reads were imputed via MAGIC [15]. Correlation analysis was performed via the WGCNA[16]. Gene ontology analysis was performed via the topGO R package[17].

The transcriptomic results of Patch-seq samples consisting of interneurons from the visual cortex was analyzed via the same pipeline seen above excluding MAGIC imputation[18]. Data consisted of 4,435 cells of which 777 were labeled as PV interneurons, and 271 as SNCG interneurons. Analysis of the electrophysiology of the associated cells began with identification of the rheobase threshold, and identification of cell latency and average firing rate at 30-40 mV above the rheobase threshold (depending on data availability). Statistical results were computed using the Wilcoxon rank sum test. PV+/CCK+ and PV+/CCK- were identified as the PV interneurons showing CCK expression in the top (bottom) 25% of PV.

Statistical Analysis

Data were presented as mean ± S.E.M. unless otherwise indicated. For statistical comparisons between groups, the data were checked for normality distribution and equal variance. If the criteria where met, a t test was performed to compare two groups; when the criteria were not met, a Mann-Whitney U-test was used. For statistical comparisons across more than two groups, we used One-Way ANOVA or the Kruskal–Wallis test (non-parametric One-Way ANOVA) and post hoc comparison tests for group comparisons. In all experiments, the level of statistical significance was defined as $p < 0.05$.

1 Taniguchi, H. *et al.* A resource of Cre driver lines for genetic targeting of GABAergic neurons in cerebral cortex. *Neuron* **71**, 995-1013, doi:10.1016/j.neuron.2011.07.026 (2011).
2 Miyoshi, G. *et al.* Genetic fate mapping reveals that the caudal ganglionic eminence produces a large and diverse population of superficial cortical interneurons. *J Neurosci* **30**, 1582-1594, doi:10.1523/JNEUROSCI.4515-09.2010 (2010).
3 Sousa, V. H., Miyoshi, G., Hjerling-Leffler, J., Karayannis, T. & Fishell, G. Characterization of Nkx6-2-derived neocortical interneuron lineages. *Cereb Cortex* **19 Suppl 1**, i1-10, doi:10.1093/cercor/bhp038 (2009).
4 Hippenmeyer, S. *et al.* A developmental switch in the response of DRG neurons to ETS transcription factor signaling. *PLoS Biol* **3**, e159, doi:10.1371/journal.pbio.0030159 (2005).
5 Madisen, L. *et al.* A robust and high-throughput Cre reporting and characterization system for the whole mouse brain. *Nat Neurosci* **13**, 133-140, doi:10.1038/nn.2467 (2010).
6 Mo, A. *et al.* Epigenomic Signatures of Neuronal Diversity in the Mammalian Brain. *Neuron* **86**, 1369-1384, doi:10.1016/j.neuron.2015.05.018 (2015).
7 Xu, X., Roby, K. D. & Callaway, E. M. Immunochemical characterization of inhibitory mouse cortical neurons: three chemically distinct classes of inhibitory cells. *J Comp Neurol* **518**, 389-404, doi:10.1002/cne.22229 (2010).
8 Jing, D. *et al.* Tissue clearing of both hard and soft tissue organs with the PEGASOS method. *Cell Res* **28**, 803-818, doi:10.1038/s41422-018-0049-z (2018).
9 Yi, Y. *et al.* Mapping of individual sensory nerve axons from digits to spinal cord with the Transparent Embedding Solvent System. *bioRxiv*, 2021.2011.2013.467610, doi:10.1101/2021.11.13.467610 (2021).
10 Renier, N. *et al.* iDISCO: a simple, rapid method to immunolabel large tissue samples for volume imaging. *Cell* **159**, 896-910, doi:10.1016/j.cell.2014.10.010 (2014).
11 Xu, X. *et al.* High-resolution and cell-type-specific photostimulation mapping shows weak excitatory vs. strong inhibitory inputs in the bed nucleus of the stria terminalis. *J Neurophysiol* **115**, 3204-3216, doi:10.1152/jn.01148.2015 (2016).
12 Yao, Z. *et al.* A taxonomy of transcriptomic cell types across the isocortex and hippocampal formation. *Cell* **184**, 3222-3241.e3226, doi:10.1016/j.cell.2021.04.021 (2021).
13 Stuart, T. *et al.* Comprehensive Integration of Single-Cell Data. *Cell* **177**, 1888-1902.e1821, doi:10.1016/j.cell.2019.05.031 (2019).
14 Korsunsky, I. *et al.* Fast, sensitive and accurate integration of single-cell data with Harmony. *Nat Methods* **16**, 1289-1296, doi:10.1038/s41592-019-0619-0 (2019).
15 van Dijk, D. *et al.* Recovering Gene Interactions from Single-Cell Data Using Data Diffusion. *Cell* **174**, 716-729.e727, doi:10.1016/j.cell.2018.05.061 (2018).
16 Langfelder, P. & Horvath, S. WGCNA: an R package for weighted correlation network analysis. *BMC Bioinformatics* **9**, 559, doi:10.1186/1471-2105-9-559 (2008).
17 Alexa, A. & Rahnenfuhrer, J. topGO: enrichment analysis for gene ontology.
18 Gouwens, N. W. *et al.* Integrated Morphoelectric and Transcriptomic Classification of Cortical GABAergic Cells. *Cell* **183**, 935-953.e919, doi:10.1016/j.cell.2020.09.057 (2020).
